# Rapid and high-purity differentiation of human medium spiny neurons reveals LMNB1 hypofunction and subtype necessity in modeling Huntington’s disease

**DOI:** 10.1186/s41232-024-00320-x

**Published:** 2024-02-15

**Authors:** Junjiao Wu, Jie Ren, Hongfei Cui, Yali Xie, Yu Tang

**Affiliations:** 1 Department of Rheumatology and Immunology, Xiangya Hospital, Central South University, Changsha, 410008 China; 2 Provincial Clinical Research Center for Rheumatic and Immunologic Diseases, Xiangya Hospital, Central South University, Changsha, 410008 China; 3 Department of Geriatrics, National Clinical Research Center for Geriatric Disorders, Xiangya Hospital, Central South University, Changsha, 410008 Hunan China; 4 Department of Neurology, Xiangya Hospital, Central South University, Changsha, Hunan, 410008 China; 5 Key Laboratory of Hunan Province in Neurodegenerative Disorders, Central South University, Changsha, Hunan, 410008 China

**Keywords:** Huntington’s disease, Medium spiny neuron, Human pluripotent stem cells, Nucleocytoplasmic transport, LMNB1

## Abstract

**Background:**

Different neural subtypes are selectively lost in diverse neurodegenerative diseases. Huntington’s disease (HD) is an inherited neurodegenerative disease characterized by motor abnormalities that primarily affect the striatum. The Huntingtin (HTT) mutation involves an expanded CAG repeat, leading to insoluble polyQ, which renders GABA^+^ medium spiny neurons (MSN) more venerable to cell death. Human pluripotent stem cells (hPSCs) technology allows for the construction of disease-specific models, providing valuable cellular models for studying pathogenesis, drug screening, and high-throughput analysis.

**Methods:**

In this study, we established a method that allows for rapid and efficient generation of MSNs (> 90%) within 21 days from hPSC-derived neural progenitor cells, by introducing a specific combination of transcription factors.

**Results:**

We efficiently induced several neural subtypes, in parallel, based on the same cell source, and revealed that, compared to other neural subtypes, MSNs exhibited higher polyQ aggregation propensity and overexpression toxicity, more severe dysfunction in BDNF/TrkB signaling, greater susceptibility to BDNF withdrawal, and more severe disturbances in nucleocytoplasmic transport (NCT). We further found that the nuclear lamina protein LMNB1 was greatly reduced in HD neurons and mislocalized to the cytoplasm and axons. Knockdown of HTT or treatment with KPT335, an orally selective inhibitor of nuclear export (SINE), effectively attenuated the pathological phenotypes and alleviated neuronal death caused by BDNF withdrawal.

**Conclusions:**

This study thus establishes an effective method for obtaining MSNs and underscores the necessity of using high-purity MSNs to study HD pathogenesis, especially the MSN-selective vulnerability.

**Supplementary Information:**

The online version contains supplementary material available at 10.1186/s41232-024-00320-x.

## Introduction

Neurodegenerative diseases are caused by the loss of neurons and disruption of neural circuits in the brain. Common neurodegenerative diseases include Alzheimer’s disease (AD), Parkinson’s disease (PD), Huntington’s disease (HD), amyotrophic lateral sclerosis (ALS), and spinal muscular atrophy (SMA), among others. The types of damaged neurons are not the same in different neurodegenerative diseases, e.g., PD mainly involves damage to dopaminergic neurons (DA), ALS and SMA mainly involve damage to motor neurons (MN), and HD primarily involves damage to medium-sized spiny neurons (MSN). Notably, the mechanism by which selective damage to specific neurons is yet clear.

HD is an autosomal dominantly inherited neurodegenerative disorder with motor abnormalities and altered mental status [1]. The lesions of HD are predominantly in the striatum, followed by the medial pallidum and cerebral cortex, thalamus, and hypothalamus [2]. Patients with HD have increased ventricular volume with a corresponding decrease in total brain volume, and varied rates of neuronal loss among brain regions [3, 4]. Imaging findings show progressive atrophy of the basal ganglia in symptomatic HD patients and pre-symptomatic patients [5]. In patients with severe HD, the weight of the caudate nucleus, shell nucleus, and pallidum was reduced by an average of 60%.

The Huntingtin gene (HTT) encoded a protein that contains a polyglutamine (polyQ) sequence at its N-terminus, which is derived from the transcriptionally encoded CAG repeat sequence. In normal populations, the CAG sequence usually has 6 to 35 repeats, and its encoded polyQ is soluble; but when there are more than 35 such repeats (mutant HTT; mHTT), the probability of HD onset increases with the number of repeats. mHTT, which encodes insoluble polyQ, renders particularly susceptible to cell death in γ-aminobutyric acid (GABA)-positive MSN neurons in the striatum [6].

Specific acquisition of MSN neurons is an important prerequisite for studying their selective death in HD. Currently, there are several animal models to characterize HD, including the widely used mice model [7–11], the Drosophila model [12] or the recently established pig model [13]. However, it is worth noting that the life cycle, physiology, and disease etiology of rodents and humans are still very different [14], which makes the translation of previous research findings inefficient. Therefore, HD research still requires reliable human models, especially patient-derived cellular models, to mimic the pathological changes of the disease and to serve as a better complement to animal models.

Over the past decade, the rapid development of human pluripotent stem cells (hPSCs), including human embryonic stem cells (hESCs) and human-induced pluripotent stem cells (hiPSCs), has brought new hope for the study of neurological diseases. In particular, hiPSCs derived from patient’s somatic cells can be used to construct a wide range of disease-specific models, including HD [15]. With the potential for unlimited proliferation, self-renewal and multi-directional differentiation, hPSCs can differentiate into neural precursor cells (NPCs) and further into neurons and glial cells, providing an important cellular model for studying disease pathogenesis, screening of disease markers and drugs, high-throughput analysis, as well as cell transplantation [16, 17].

Although hPSCs provide a large source of cells, efficient generation of diverse neuron subtypes for different neurodegenerative diseases remains a major challenge. Neural differentiation generally follows neurodevelopmental timing and regulatory factors, including transcription factors or non-coding RNAs (e.g., miRNAs), and induction of signaling molecules such as cytokines (e.g., SHH and WNT). Traditional methods of neural differentiation, such as induction and picking of neural rosettes and multi-step chronological addition of cytokines have resulted in the generation of various neural subtypes, including MSNs, but with high variability or excessive duration of differentiation [18–23]. To facilitate the clinical use, attempts have also been devoted to generate clinical-grade MSNs by introducing the γ-secretase inhibitor DAPT and small molecules, instead of protein components, into the differentiation recipe [24].

In recent years, it has become possible to directly reprogram somatic cells (e.g., fibroblasts and astrocytes) into progenitor cells (e.g., neural stem cells) or other differentiated cells (e.g., neurons) using specific transcription factors [25–27]. Transcription factors bind to specific DNA motifs to alter gene expression and further the state of a particular cell. Notably, transcription factors involved in cell fate transitions have recently been systematically studied [28–30]. Similarly, studies have been conducted to apply transcription factors to the neural differentiation of hPSCs to differentiate hPSCs into specific neural subtypes, and these approaches have the advantage of efficiently and rapidly generating high-purity cell populations such as glutamatergic neurons, MN, DA, and GABA [31–36]. However, studies using specific transcription factors to effectively induce MSN generation have not been reported.

Proneural factors include transcription factors encoding the bHLH class, which are primarily responsible for the development of neural ectodermal progenitor cells. Currently widely used transcription factors or miRNAs include ASCL1, NGN2, NeuroD1, miR-9/124, and inhibition of PTBP1 [27]. Neural differentiation can be rapidly induced by proneural factors, while for specific neural subtypes, proneural factors alone are not sufficient and extra transcription factors related to the lineages of neuronal subtypes need to be added. For instance, in the MN induction method previously reported by our group, the proneural factor NGN2 combined with the MN lineage-related ISL1/LHX3 can efficiently induce MN production [37–39]. Therefore, similar strategies can be used to differentiate other neural subtypes such as MSN.

To efficiently induce hPSCs toward MSNs, we probed a specific combination of transcription factors that can rapidly and efficiently generate MSNs by testing the neural induction ability of different proneural factors, as well as MSN lineage factors. We simultaneously induce MSN, MN, DA, and DARPP32(-) GABA (hereafter refers to GABA neurons) neurons from the starting cells, and showed that MSN mimics the pathological features of HD more readily than other neural subtypes in a number of ways. Notably, we further found that the nuclear lamina protein LMNB1 was greatly downregulated and mislocalized to the cytoplasm in HD neurons. Knockdown of HTT or treatment with KPT335, an orally selective inhibitor of nuclear export (SINE), effectively attenuated the pathological phenotypes and alleviated neuronal death caused by BDNF withdrawal. This study establishes an efficient and rapid method to obtain MSNs and demonstrates the importance and necessity of using high-purity MSNs to model HD pathogenesis, especially the MSN-selective vulnerability.

## Materials and methods

### Cell lines and culture conditions

H9 ESC line (WA09) was obtained from the WiCell Research Institute. The HD iPSC line (GM23225), heterozygous for HTT^Q19/Q72^, was purchased from the Coriell Institute for Medical Research. hPSCs were maintained in daily-replaced ncTarget medium (Nuwacell, China) on Matrigel (Corning)-coated plates at 37 °C and 5% CO_2_. Cells were routinely passaged upon ~ 80% confluency using Versene (Gibco). 293 T and A375 cells were cultured in DMEM (Gibco) containing 10% fetal bovine serum (FBS) (TransGen Bio, China) and 1% penicillin/streptomycin (P/S).

### Vector constructions

The coding sequences (CDS) of major proneural factors including NGN2, ASCL1, NeuroD1, and miR-9/124, as well as MSN lineage factors, were individually cloned into a third-generation lentiviral vector (pCSC-pTetO-ires-GFP). For the combinations of transcription factors, two lineage factors were linked with 2A peptide to ensure an equivalent expression level. In several cases, the proneural factor and two lineage factors were cloned into one single vector, to further enhance the efficiency. PolyQ25::GFP, polyQ46::GFP and polyQ97::GFP were constructed into lentiviral vectors containing the CMV promoter or the synapsin promoter (pCSC-pCMV-ires-puro; pCSC-pSyn-ires-puro), respectively.

The pLKO.1 lentiviral vector was applied to express LMNB1 shRNAs. Briefly, shRNA oligos targeting the coding region were synthesized and ligated to the AgeI and EcoRI restriction sites of pLKO.1. The targeting sequences are:AGCTTCTTGATGTAAAGTTA (shLMNB1-1), and CAGACTGTCATCAGAGATGAA (shLMNB1-2). The vectors of lentiCRISPRv2-mCherry and lentiCRISPRv2-blast were used to express PTBP1 and HTT sgRNAs. Briefly, sgRNA oligonucleotides were designed by the CRISPOR webserver, and then synthesized and ligated into lentiCRISPRv2 vectors at the BsmBI site. The targeting sequences are CTCCGTGTTCATCTCGATGA (sgPTBP1-1), CGGGGTCCGCATACTGCAGC (sgPTBP1-2), GCTTTTCCAGGGTCGCCATGG (sgHTT-1), and GTGCCGGGCGGGAGACCGCCA (sgHTT-2). All vectors were verified by restriction digest and Sanger sequencing.

### Virus preparation

Each of lentiviral vectors was co-transfected with two packaging vectors (psPAX2, pMD2.G) into 293 T cells using polyethylenimine (PEI) reagent. Viral supernatants were respectively collected 48 and 72 h post-transfection and filtered by 0.45 µm syringe filters. Lentiviruses were concentrated with PEG8000 and stored as aliquots at – 80 ℃ for further use.

### Genomic targeting

The T7 Endonuclease I (T7E1) assay was performed to assess the cleavage efficiency of sgRNAs. Briefly, 293 T cells were transfected with vectors containing sgRNAs for 3 days. Genomic DNAs were isolated and used as the templates for targeted PCR. The amplified DNA products were melted and re-annealed to form heteroduplexes, which were then digested with T7E1 (NEB) for 1 h. For the group of mixed sgHTT-1/2, the amplified DNA product was digested with NcoI, which is localized in the vicinity of sgHTT target sites. All digested products were electrophoresed in 2% agarose gels and visualized by the Gel Doc EZ Imager (Bio-Rad). The fraction of PCR product cleaved (f_cut_) was calculated using the following formula: f_cut_ = (b + c)/(a + b + c), where a is the combined intensity of the undigested PCR product, and b and c are the combined intensities of each cleavage product. Indel occurrence was estimated using the following equation: $\mathrm{indel }\left(\mathrm{\%}\right)=100\mathrm{ x }\left(1-\sqrt{\left(1-{f}_{cut}\right)}\right)$.

### NPC differentiation from hPSCs

The rtTA-N144 (addgene 66810) concentrated lentivirus was first transferred into hPSCs, followed by hygromycin screening, to eventually obtain rtTA stably-expressed hPSCs (rtTA-hPSCs). NPC generation was performed according to previously reported protocols with minor modifications [38–40]. Briefly, a total 2 × 10^6^ undifferentiated rtTA-hPSCs were passaged by Versene and seeded onto a Matrigel-coated 6-well plate. Upon over 90% confluency, cells were cultured in ncTarget medium containing 10 μM SB431542 (Sigma) and 0.1 μM LDN193189 (Miltenyi Biotec) for 7 days. Next, cells were dissociated by Versene and gently pipetted into small clumps for suspension in low-attachment 10-cm petri dishes (Grainger). Cell clumps were cultured to form neurospheres in KOSR medium supplemented with 10 µM Y-27632 for 4 days, and further cultured in NSP medium for 7 days. Neurospheres were collected in conical tubes and washed once with DPBS, followed by dissociating into single cells by Accutase (Innovative Cell Technologies). Finally, dissociated single cells were cultured and maintained in NPC medium.

The medium formulations were as follows: (a) hPSC medium: DMEM/F12 medium with 20% KnockOut Serum Replacement (KOSR; Gibco), 1% GlutaMax, 1% non-essential amino acid (NEAA), 50 μM β-Mercaptoethanol (β-ME), 1% P/S, and 10 ng/ml bFGF (PeproTech). (b) KOSR medium: hPSC medium without bFGF. (c) Neurosphere medium (NSP medium): DMEM/F12 medium containing 1% N2 (Invitrogen), 1% GlutaMax, 1% NEAA, 50 μM β-ME, 1% P/S, 8 μg/ml Heparin (Sigma), 20 ng/ml bFGF, and 20 ng/ml epidermal growth factor (EGF; PeproTech). For MSN differentiation, neurospheres were cultured with KOSR medium and NSP medium supplemented with 25 ng/ml Activin A (Sino Biological, China). (d) Neural progenitor cell medium (NPC medium): DMEM/F12 and Neurobasal medium (1:1) containing 0.5% N2, 1% B27 (Invitrogen), 1% GlutaMax, 1% NEAA, 50 μM β-ME, 1% P/S, 10 ng/ml EGF, and 10 ng/ml bFGF.

### Neural differentiation and treatments

The overall schedule of neural differentiation from hNPCs is list in Fig. S1. Briefly, rtTA-hNPCs were seeded at a density of 3 × 10^4^ cells/cm^2^ onto Matrigel (1:500)/Laminin (5 μg/ml)/Fibronectin (5 μg/ml)-coated plates and grown in NPC medium. The next day, rtTA-hNPCs were infected with a single dose or a cocktail of lentiviruses at 50 MOI expressing proneural factors or MSN lineage factors, supplemented with 6 μg/ml polybrene. During differentiation, part of rtTA-hNPCs were infected with concentrated lentiviruses (pCSC-pSyn-polyQ25/46/97::GFP) with a low MOI (MOI = 3). The next day, the medium was replaced with neural differentiation medium (DMEM/F12 and Neurobasal medium (2:1) containing 0.8% N2, 0.4% B27, 0.4 µg/ml L-AA, 5 µM forskolin, 10 ng/ml BDNF, and 10 ng/ml GDNF). At the same time, cells were treated with doxycycline (1 μg/ml) to induce the expression of proneural factors and MSN lineage factors. The culture medium was routinely replaced twice a week. Doxycycline was withdrawn at 6 dpi.

Part of differentiated neurons were then dissociated by Accutase at 6 dpi, and replated onto astrocyte-coated coverslips at a density of 1 × 10^3^ cells/cm^2^ for immunostaining, as well as the analysis of polyQ aggregations, protein transportation and mislocalization. Primary astrocytes were isolated from postnatal day 1 (P1) mouse pups according to previously described procedures [41]. Endogenous mouse neurons were removed by a few cycles of passaging, freezing, thawing, and plating. Notably, during the differentiation process, puromycin (0.5 μg/ml) and AraC (1 μM) can be used respectively to treat cells for 24 h, to inhibit the growth of differentiated glia cells and improve the purity of neurons. Neurons were routinely cultured in C2 medium until analysis. To test the propensity of polyQ aggregation, differentiated neurons were treated with MG132 (10 μM) for 24 h for further immunostaining analysis.

For the differentiation of pan-neurons, NPCs were digested with Accutase and seeded onto Matrigel (1:500)/Laminin (5 μg/ml)/Fibronectin (5 μg/ml)-coated plates at a density of 3 × 10^4^ cells/cm^2^ and cultured with C2 medium. The medium was half-replaced every other day until analysis.

### Assessment of neural development

During the process of neural differentiation, neurons were fixed with 4% paraformaldehyde (PFA) at desired time points to observe and count the number of neurons, and to calculate neurite length and number of branches as well as soma sizes. Neurite lengths were measured with the ImageJ software and expressed as the relative length of each neuron. Neurons directly connected to the soma were counted and shown as major branches for each cell. The maturity of neurons was assessed by Sholl analysis.

### NCT detection and treatment

The concentrated lentivirus pLVX-2xGFP::NES-Ires-2xRFP::NLS (2Gi2R) with a low MOI (MOI = 3) was added to infect differentiated neurons at 21 dpi for overnight, and fresh C2 medium was replaced the next day. After 3 days, neurons were fixed with 4% PFA at room temperature for 20 min. GFP and RFP signals were evaluated under a confocal microscope (Zeiss LSM710) and analyzed with ImageJ. The LMNB1 mislocalization was assessed by immunostaining in differentiated neurons at desired time points (14, 21, and 30 dpi). Nuclei were stained with DAPI to distinguish between nucleus and cytoplasm.

### BDNF withdrawal and neural survival

Forskolin was removed from C2 medium before starting the assay, as it might increase the endogenous BDNF expression. At 21 dpi, neurons were cultured with C2 medium containing BDNF (100 ng/ml) for 24 h, followed by an acute withdrawal of BDNF (0 ng/ml) for another 24 h. To target the NCT disturbance, neurons were pre-treated with KPT335 (25 nM or 50 nM) for 24 h prior to withdrawal of BDNF. Neuronal death was assessed by Annexin V staining, according to the manufacturer’s instructions (Beyotime, China).

### Gene expression

To detect the expression of neural markers, BDNF signaling genes, and HD-affected genes by quantitative PCR, neurons were harvested with TRIzol at desired time points for RNA isolation. Briefly, total mRNAs were extracted and reverse-transcribed into cDNAs using with the SuperScriptIII First-Strand kit (Life Technologies). Quantitative PCR was performed using TransStart Green qPCR SuperMix (TransGen Bio, China) and carried out on a 96-well QuantStudio Dx thermocycler (Applied Biosystems). All primers were verified by melting curve analysis with a single melt curve peak. Relative expression levels of each gene were analyzed using the 2^−∆∆Ct^ algorithm, normalized to the expression levels of the housekeeping gene HPRT and relative to control samples. Primer sequences are listed in Table S1.

### Immunostaining

Cultured neurons at desired time points were fixed with 4% PFA at room temperature for 20 min. After a brief rinse with PBS, cells were permeabilized and blocked with blocking buffer (PBS containing 0.2% Triton X-100 and 3% BSA) for 30 min. They were then incubated with primary antibodies in blocking buffer at 4 °C for overnight, followed by repeated washing and incubation with corresponding Alexa Fluor-conjugated secondary antibodies (Invitrogen, 1:500) for 1 h at room temperature. Cell nuclei were counter-stained with DAPI. Confocal images were obtained and analyzed using a confocal microscope (Zeiss LSM710). The primary antibodies used are shown in Table S2.

### Immunoblot

Cultured neurons at desired time points were lysed in RIPA lysis buffer (Beyotime), supplemented with protease inhibitor cocktail (TransGen Bio). Equal amounts of cell lysates (30 μg per lane) were used for running SDS-PAGE. Briefly, proteins were separated by SDS-PAGE with Tris–glycine gels and transferred onto PVDF membranes (Millipore). Non-specific sites were blocked in 5% skimmed milk for 1 h at room temperature. The blots were then incubated at 4°at room temperature. The blots were then incubated at 4teins were separated by SDS-PAGE wi3 times, and incubated with secondary antibodies conjugated with horseradish peroxidase (1:5000; Servicebio, China) at room temperature for 1 h. Finally, protein bands were visualized with ECL substrate (TransGen Bio) and ChemiDoc XRS + Imager (Bio-Rad). Primary antibodies used are shown in Table S2.

### Statistical analysis

Data were expressed as the mean ± SEM values of at least three biological replicates. Two-tailed student’s *t* test or two-way ANOVA were performed with GraphPad Prism to assess the statistical significance among groups. Differences with *p* values less than 0.05 were considered to be significant.

## Results

### Proneural factors for inducing neural morphologies

**Fig. 1:**
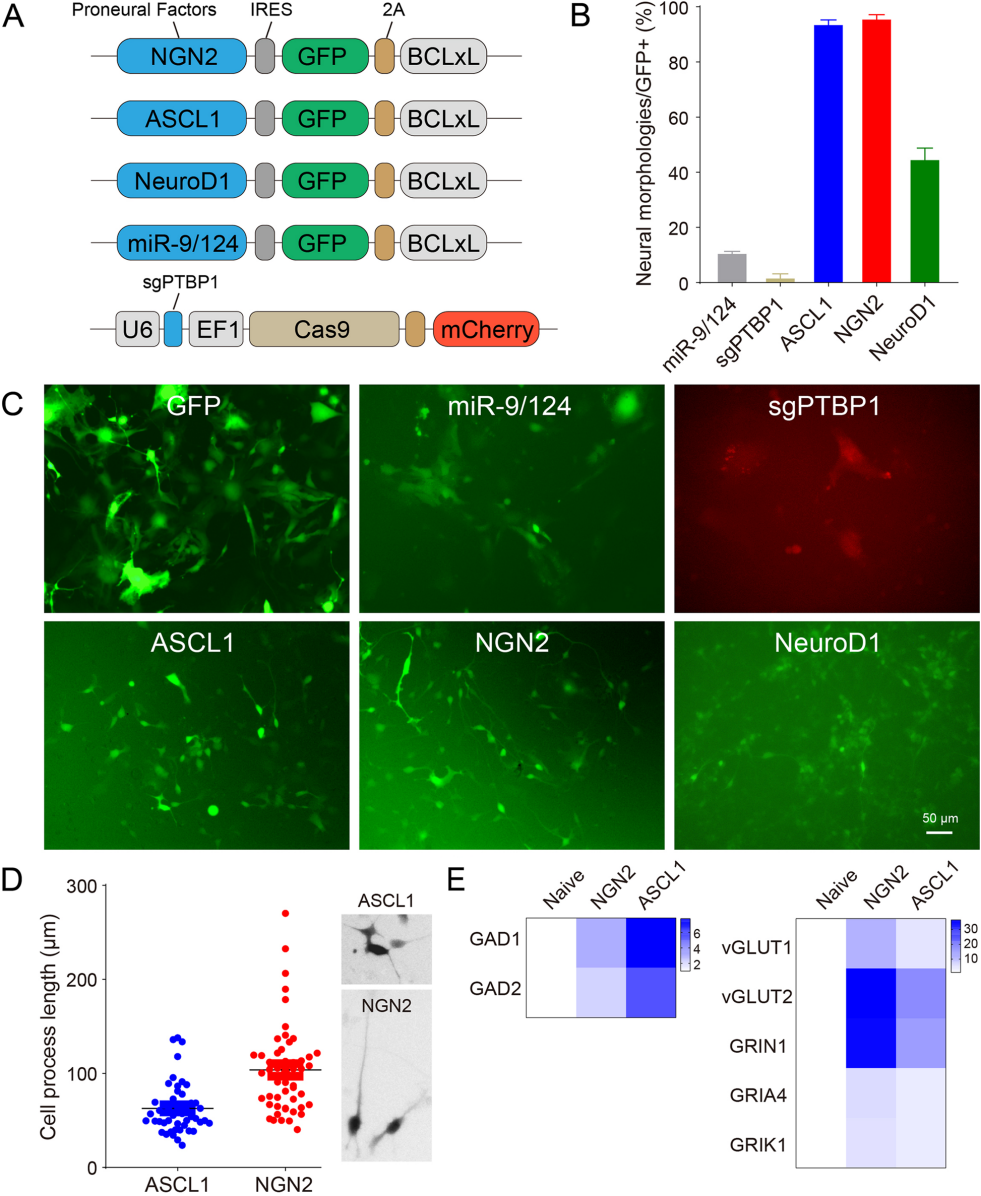
The capacities of neural induction of proneural factors.** A** Typical proneural factors, including NGN2, ASCL1, NeuroD1, and miR-9/124, as well as the knockdown of PTBP1 by sgPTBP1, were respectively constructed into lentivirus vectors. **B**, **C** The capacities of neural induction of proneural factors were examined. NGN2 and ASCL1 contributed to over 90% changes into neural morphologies at 3 dpi, whereas miR-9/124 and sgPTBP1 barely induced changes. Scale bar, 50 μm. **D** The cell processes of induced cells were better, or at least faster, induced by NGN2, rather than by ASCL1. **E** Quantitative expression analysis of the induced cells. NGN2 mainly induced the generation of excitatory neurons (vGLUT1, vGLUT2, GRIN1, GRIA4 and GRIK1), whereas ASCL1 mainly induced the generation of inhibitory neurons (GAD1 and GAD2)

Typical proneural factors, including NGN2, ASCL1, NeuroD1, and miR-9/124, as well as the knockdown of PTBP1 by Cas9/short-guide RNAs (sgRNAs), are widely used in both hPSC programming and direct reprogramming toward neurons [42] (Fig. 1A). To test their capacities of neural induction in our context, we constructed them as viruses and infected NPCs. Based on the neural-like morphologies at an early stage of 3 dpi, we revealed that NGN2 and ASCL1 contributed to over 90% morphology changes, whereas miR-9/124 and sgPTBP1 (Fig. S2A) barely induced the changes (Fig. 1B, C). The results of the role of miR-9/124 and sgPTBP1 in initiating neural conversions in our study were unexpected, as they have been well-documented to promote neuronal lineage conversion in multiple studies [43]. Mechanistically, miR-124 and PTBP1 act synergistically as a regulatory loop [44]. Overexpression of miR-9/124 in human fibroblasts attenuates the expression of negative regulators of neurogenesis (e.g., PTBP1) by triggering extensive remodeling of the chromatin accessibility landscape [45, 46]. On the other hand, PTBP1 acts as an RNA-binding protein that regulates mRNA splicing and prevents the activation of miRNAs (e.g., miR-124) by competitively binding to or altering the mRNA secondary structure [47]. It might be possible that the epigenetic landscape of our NPCs is more resembling a neural lineage than that of fibroblasts or astrocytes, rendering miR-9/124 and sgPTBP1 refractory to a fierce neural conversion.

Furthermore, we also observed that the cell processes were better, or at least faster, induced by NGN2, rather than by ASCL1 (Fig. 1D). This is also consistent with previous studies that have shown that NGN2 overexpression generates mature neurons faster than ASCL1 overexpression, highlighting their different kinetics in neural conversions [48]. This may be due to the intrinsic effects of NGN2 in initiating cellular polarity, as we tested this in non-neural A375 cells and observed that NGN2 also triggered a morphology similar to neural processes (Fig. S3).

Quantitative expression analysis showed that NGN2 primarily induced excitatory neuron generation as assessed by vGLUT1, vGLUT2, GRIN1, GRIA4, and GRIK1, whereas ASCL1 mainly induced inhibitory neuron generation as assessed by GAD1 and GAD2 (Fig. 1E). This is in line with the previous knowledge that NGN2 and ASCL1 are expressed in a relative complementary manner in the telencephalon: NGN2 is expressed in dorsal progenitors, instructing them to generate glutamatergic neurons, while ASCL1 is expressed in ventral progenitors, facilitating the acquisition of GABAergic neuronal fates [42, 49].

### Generation of MSNs by adding MSN lineage factors

Given that MSNs originate from lateral ganglionic eminence (LGE) located in the ventral region during neural development and are bona fide GABAergic neurons, ASCL1 was thus selected as the optimal proneural factor for inducing MSNs. However, ASCL1 alone did not induce the expression of DARPP32, the major maker of MSN, and additional lineage factors were required (Fig. S4). Notably, previous studies have directly applied MSN lineage factors, including ISL1/ZNF503 and GSX2/EBF1 [50], without adding proneural factors, to induce MSNs. We thus also tested them but found that these two groups were not effective in inducing neural morphologies (Fig. S2B, C).

We next searched for additional lineage factors that could synergize with ASCL1. We made an initial attempt by testing different combinations of ASCL1 and 13 selected lineage factors, and further tested the DARPP32 expression (Fig. S4A, B). We found that ASCL1 worked well with CTIP2 and DLX1/2 to efficiently induce DARPP32 expression, whereas neither ASCL1 nor any two of them alone did, albeit causing neural morphology changes (Fig. S4B, C). In addition, we tested to combine CTIP2 and DLX1/2 with another proneural factor, miR-9/124, but failed to initiate the neural conversion (Fig. S2D), suggesting that ASCL1/CTIP2/DLX/1/2 as a whole is required for MSN differentiation. Quantitative expression analysis showed that ASCL1/CTIP2/DLX/1/2 also induced GABAergic markers, such as GAD1 and GAD2, which was also seen in groups containing ASCL1 or DLX1/2 (Fig. S4D).

### Characterization of induced neurons

**Fig. 2:**
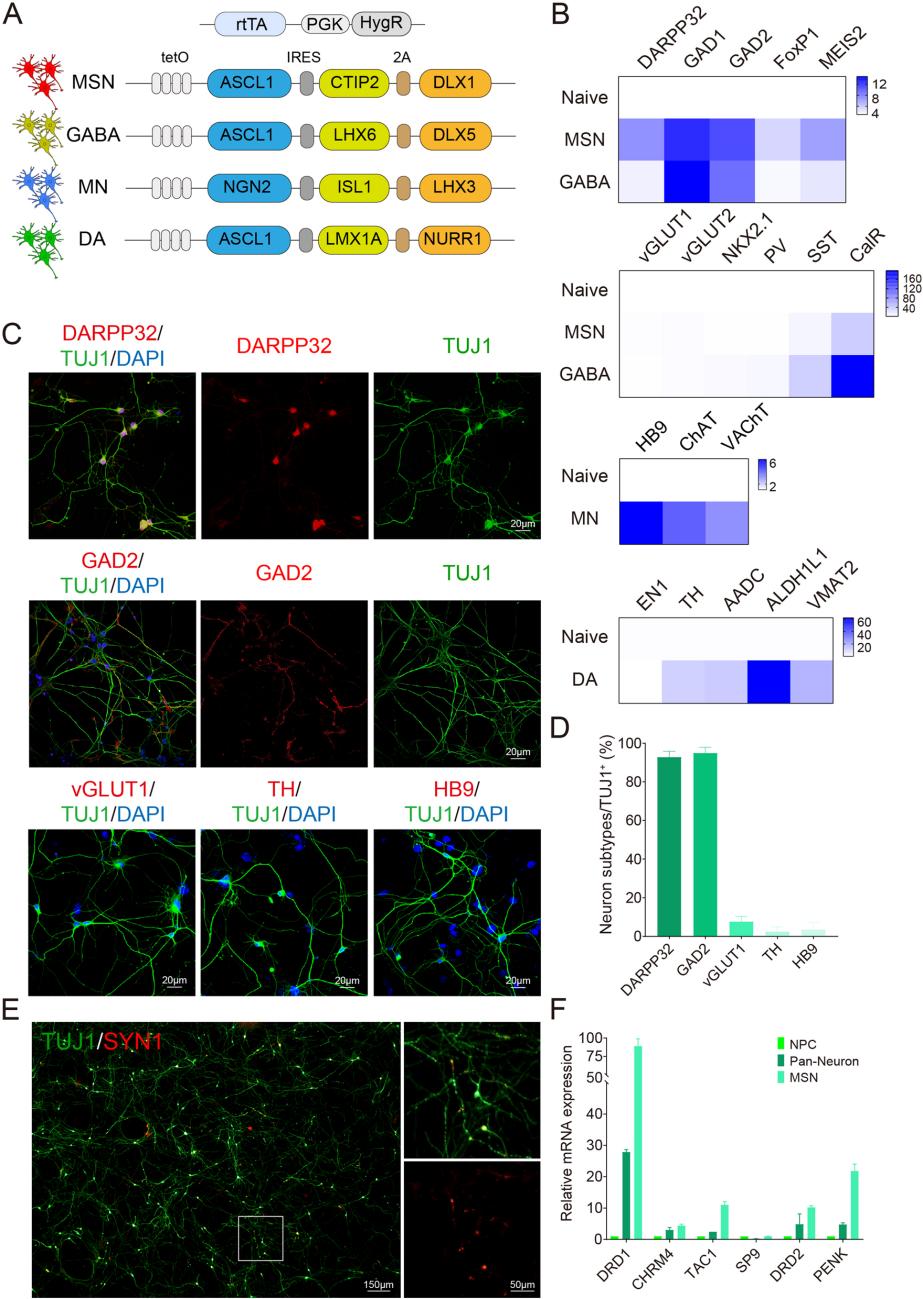
Characterization of induced neurons. **A** Four major neural subtypes including MSNs, GABA, MN, and DA were differentiated, in parallel, by induced expression of transcription factors in rtTA-expressing stable cells. **B** Neuron identities were examined by quantitative PCR of typical markers at 21 dpi. **C**, **D** Neuron identities were examined by immunostaining of typical markers at 21 dpi on the glia layer. Over 90% of TUJ1^+^ neurons were positive for MSN markers including DARPP32 and GAD2, and were devoid of other subtype markers such as vGLUT1, TH, and HB9. Scale bars, 20 μm. **E** Synapse formation was examined by immunostaining of SYN1. Scale bar, 150 μm. Inset scale bar, 50 μm. **F** Detection of MSN subtypes by quantitative PCR. MSN subtype markers were all expressed at higher levels in generated MSNs than those of pan-neurons

Next, we used the screened transcription factors to induce MSNs, while inducing other subtypes, such as MN, DA, and GABA neurons, with reported sets of transcription factors in a similar fashion (Fig. 2A). To achieve the maximal conversion efficiency, all transcription factors including proneural factors and lineage factors were engineered into one single vector, inside which, lineage factors were linked with 2A to ensure equivalent expression levels (Fig. 2A). Lentiviruses were then packaged and infected with NPCs stably expressing rtTA. To differentiate all neural subtypes in parallel, NPCs differentiation was initiated by Doxycycline treatment, while replacing with neural induction medium containing various cytokine factors.

Within 21 days, neuron identities were examined by quantitative PCR and immunostaining. Differentiated MSNs expressed high levels of DARPP32, whereas GABA neurons expressed only basal levels (Fig. 2B). Both MSNs and GABA neurons expressed high levels of GAD1 and GAD2, whereas vGLUT levels were extremely low (Fig. 2B). The LGE lineage marker MEIS2 was expressed at high levels in MSNs, whereas markers exclusive to striatal or cortical GABAergic interneurons, such as PV, SST, and CalR, were expressed at low levels in MSNs but at high levels in differentiated GABA neurons (Fig. 2B). In addition, the expression of NKX2.1, the typical transcriptional marker for medial ganglionic eminence (MGE), was low (Fig. 2B). Thus, these expression features allowed us to distinguish the generated MSNs from GABA neurons.

Upon immunostaining, we revealed that over 90% of TUJ1^+^ neurons were positive for MSN markers, including DARPP32 and GAD2, and were devoid of other subtype markers such as vGLUT1, TH and HB9 (Fig. 2C, D). The generated MSNs were able to form mature connections as they can form synapses (Fig. 2E). Notably, the efficiency of MSN differentiation was not compromised, when starting from HD-NPCs (Fig. S5A). Using the same differentiation strategy, other neural subtypes including GABA neurons, MN neurons, and DA neurons were also obtained as previously reported [34, 36, 39, 51]; more than 80% of these neurons were positive for GAD2, HB9/ChAT, and TH, respectively (Fig. S5B-E). They also expressed typical markers of neural subtypes, for instance, ChAT and VAChT in MN neurons, and TH, AADC, ALDH1L1, and VMAT2 in DA neurons (Fig. 2B).

To further characterize the subtypes of generated MSNs, we also differentiated NPCs toward pan-neurons (mixed neuron populations without directed differentiation into specific subtypes), as a control, without adding transcription factors (Fig. S5F). MSN subtype markers were all expressed at higher levels in generated MSNs than in pan-neurons (Fig. 2F). This differentiation strategy induced both MSN subtypes, as the expression levels of D1 striatonigral subtype markers (DRD1, CHRM4, and TAC1) and D2 striatopallidal subtype markers (DRD2, SP9, and PENK) were all effectively elevated (Fig. 2F).

### MSNs were more vulnerable to polyQ-overexpression caused damages

**Fig. 3:**
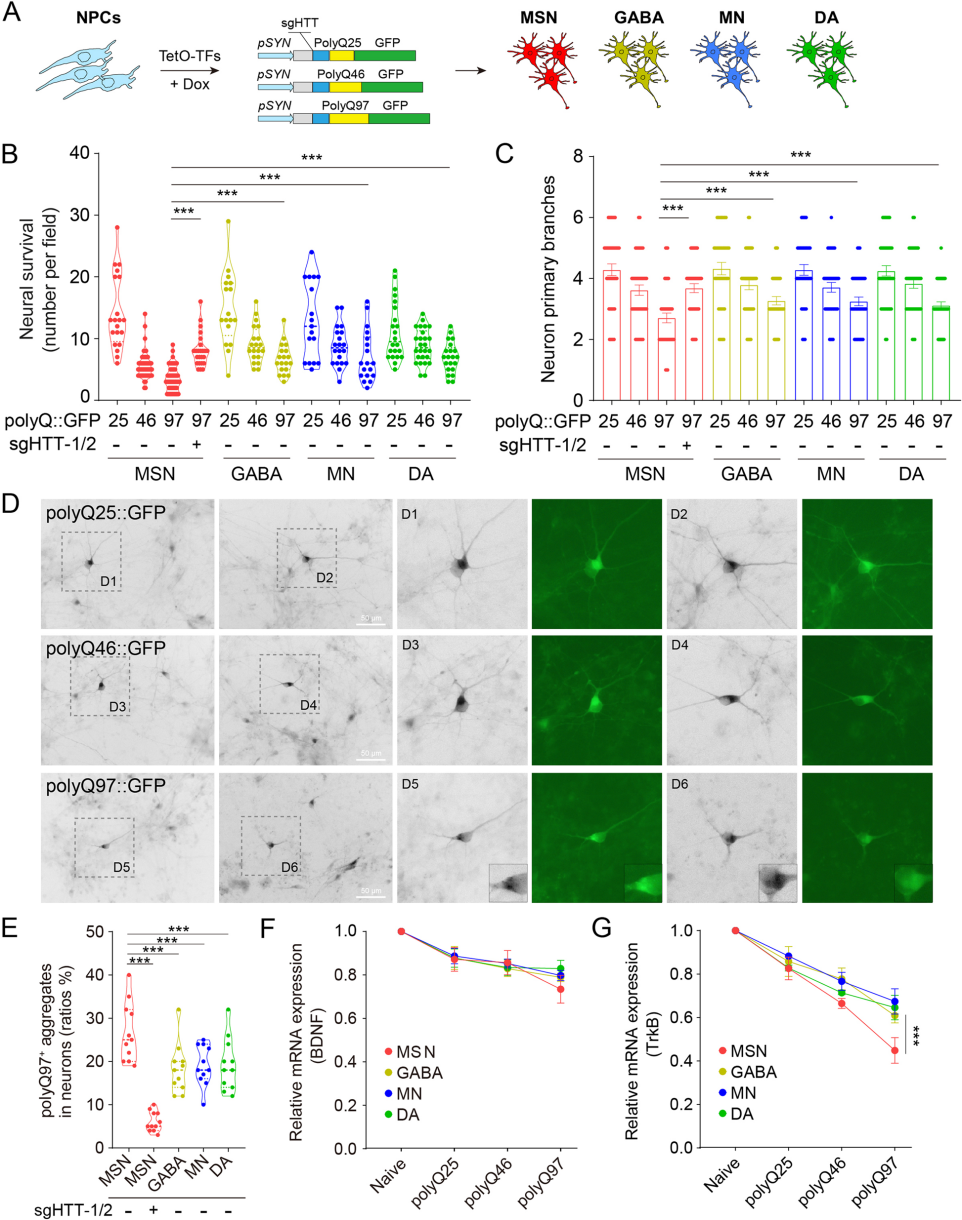
MSNs were more vulnerable to polyQ-overexpression caused damages.** A** To carry out a polyQ overexpression assay in induced neurons, lentiviruses containing different lengths of polyQs::GFP (Q25, Q46, Q97) driven by the human synapsin promoter were generated. NPCs were infected with low MOI of viruses during differentiation. **B**, **C** The overexpression was able to evoke neuronal damages, featured by reduced neural survival and primary branch numbers, in a polyQ-length dependent manner, which was ameliorated by HTT knockdown. **D**, **E** PolyQ97::GFP aggregates were observed in neurons on the glia layer, particularly prominent in MSNs, albeit to a lesser extent than at high MOI overexpression. Scale bars, 50 μm. **F**, **G** Expression levels of TrkB and BDNF were detected by quantitative PCR. ***p < 0.001

To assess the functional properties of generated MSNs, we applied a polyQ overexpression assay. Specifically, we infected neurons with viruses containing different lengths of polyQs::GFP (Q25, Q46, Q97), driven by the human synapsin promoter (Fig. 3A). We cautiously avoided to use high multiplicity of infection (MOI) of viruses, because polyQ overexpression led to dramatic neuron death within 7 days (Fig. S6). However, reduced amounts of polyQ overexpression were already able to evoke neuronal damages in a polyQ-length dependent manner, characterized by reduced neural survival and primary branches (Fig. 3B–D). PolyQ97::GFP aggregates were also observed in neurons, albeit in a milder manner than high MOI overexpression (Fig. 3D, E). Notably, the neuronal damages and polyQ aggregates were particularly prominent in MSNs, compared to other subtypes (Fig. 3B–E).

To ameliorate the polyQ-overexpression caused impairments, we designed two sgRNAs (sgHTT-1 and sgHTT-2) that target DNAs flanking the start codon of the HTT CDS, aiming to knockdown HTT overexpression (Fig. S7A). Indeed, sgHTT-1/2 effectively cleave genomic DNAs, as assessed by T7E1 and restriction fragment length polymorphism (RFLP) assays (Fig. S7B-D). We further transfected 293 T cells with different lengths of polyQs::GFP (Q25, Q46, Q97) driven by the CMV promoter and examined the efficacy of sgHTT-1/2 in vivo, confirming that they are capable of diminishing polyQ::GFP aggregates (Fig. S7E-G). By applying sgHTT-1/2 into effect, we observed that in polyQ97 overexpressed MSNs, knockdown of HTT (including mutant HTT) greatly increased neural survival and the number of primary branches, and was expected to attenuate polyQ::GFP aggregation (Fig. 3B–E).

Deficiency of brain-derived neurotrophic factor (BDNF) signaling has been extensively investigated in HD pathology and is pivotal for striatal degeneration [52–55]. We found that TrkB, the main receptor for BDNF, was progressively downregulated in polyQ-overexpressed neurons, whereas in the polyQ97 group, MSN was distinguished from the other neural subtypes (Fig. 3F). The expression of BDNF per se, however, was not significantly changed (Fig. 3G). Taken together, our results showed that MSNs were more vulnerable to polyQ-overexpression caused neural impairments.

### Neural differentiation from HD diseased hPSCs

To further evaluate the properties of MSNs, we applied HD diseased iPSCs containing heterogeneous polyQ^19/72^ (Fig. S8A, B). Both WT- and HD-iPSCs were differentiated by dual-SMAD inhibition [38, 56], followed by suspension culture as neurospheres and eventually monolayer culture as NPCs (Fig. S8C, D). The pathological changes of HD have been manifested in generated NPCs [57]. In our case, HD-NPCs proliferated much slower than WT-NPCs (Fig. S8E). Although the yield of NPCs (more than 96% of PAX6^+^ NESTIN^+^) from the HD-iPSC was comparable to that from the WT-iPSC (Fig. S8F, G), the levels of the NPC markers such as PAX6 and NESTIN were lower in HD-NPCs (Fig. S8H).

LMNB1 is an indispensable structural component of the nuclear lamina, an intermediate filament network located beneath the inner nuclear membrane. LMNB1 plays a role in the regulation of nuclear architecture and gene expressions in a variety of physiology functions, including neural development. Given the growing attention on nuclear biology in neurodegenerative diseases, we also examined LMNB1 expression in NPCs and showed that the nuclear morphology of HD-NPCs was largely deformed, along with a decrease in nuclear circularity (Fig. S8I-K). This is actually, in part, in line with previous findings in multiple models such as transgenic HD mouse models [58], Drosophila models [59], as well as hPSC models [59], reminiscent of a common feature of HD pathology.

### MSNs recapitulated the HD pathology

**Fig. 4:**
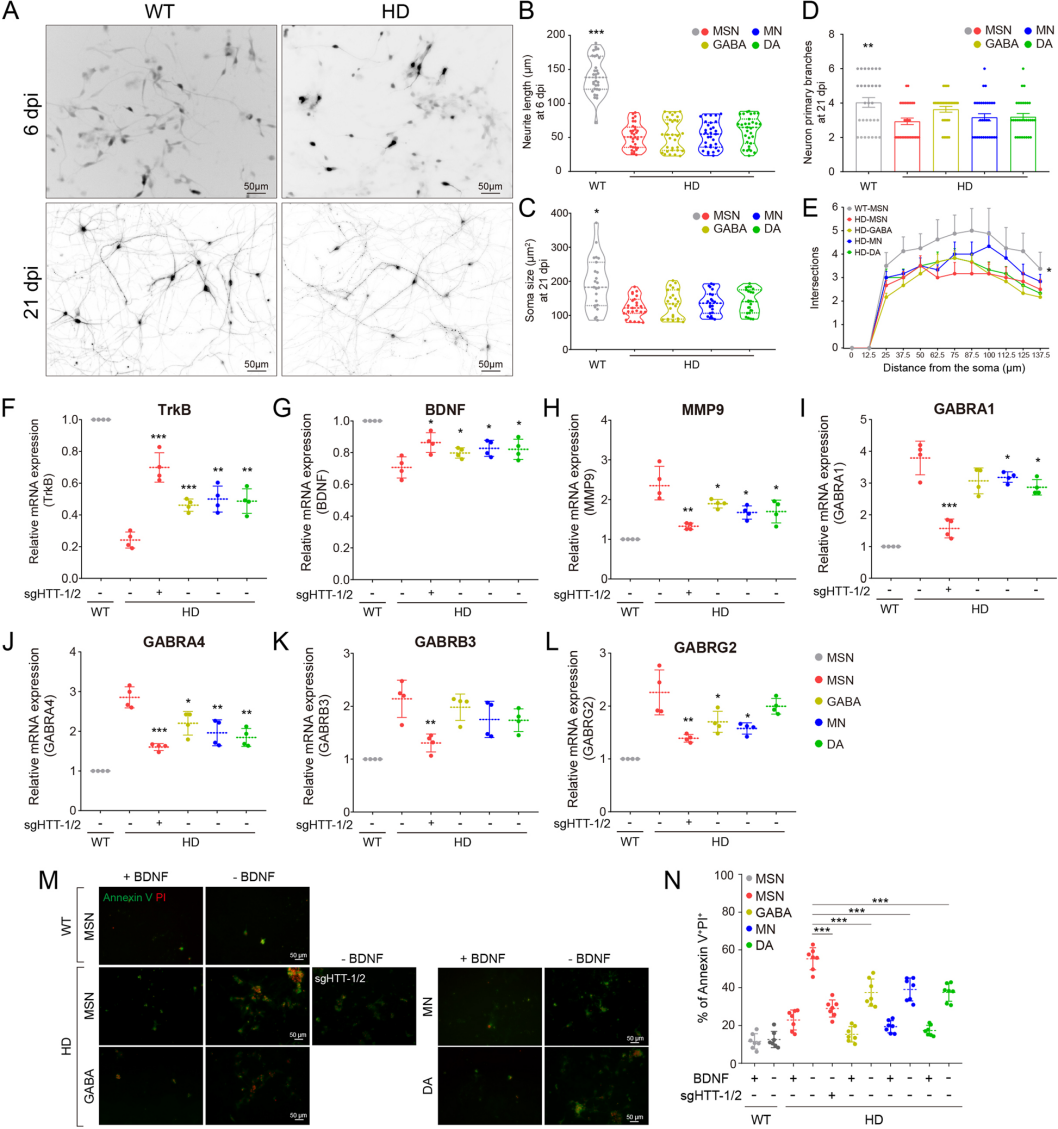
MSNs recapitulated the HD pathology. **A**–**E** Neural development in both WT and HD neurons. The neurite lengths (6 dpi), soma sizes (21 dpi), and primary branch numbers (21 dpi) were assessed, respectively. The maturation of neurons was assessed by Sholl analysis. Scale bars, 50 μm. **F**–**L** Expression of typical genes affected by HD in differentiated neurons was detected by quantitative PCR. **M**–**N** Neuronal death upon BDNF withdrawal was assessed by Annexin V staining. Scale bars, 50 μm. *p < 0.05; **p < 0.01; ***p < 0.001

To better model HD, we differentiated four major neural subtypes based on the same HD-NPCs, as well as MSN controls from WT-NPCs. We observed that the development of HD neurons was significantly slowed down over time, as evidenced by a decrease in neurite length, soma size, and number of primary branches, as well as an impaired neural survival rate (Fig. 4A–D; Fig. S9A). The maturation of HD neurons was also slowed down with fewer intersections after the Sholl analysis (Fig. 4E). However, the degree of developmental impairment was comparable among the four major subtypes, suggesting that mHTT might suppress the priming molecular machinery of neural induction, without cell-type specificity.

We further performed quantitative expression analysis of genes typically affected by HD in well-differentiated neurons. Similar to earlier results, TrkB expression was downregulated in HD neurons, while BDNF levels were unchanged (Fig. 4F, G). These results suggest that mHTT may induce downregulation of BDNF signaling at the receptor level of HD-MSNs. MMP9, which was found to be increased in postmortem human HD brain tissues [60], was also upregulated in HD neurons. In addition, other genes affected by HD, such as GABRA1, GABRA4, GABRB3, and GABRG2, were upregulated in HD neurons at the expression level (Fig. 4H–L). Of the four major neural subtypes, MSN was the most affected, which is different from comparing those developmental impairments. Furthermore, knockdown of HTT using sgHTT-1/2 significantly alleviated these abnormal expressions (Fig. 4F–L).

In mouse models of HD, damage to TrkB receptors has been shown to mediate postsynaptic dysfunction in the MSN [61]. Given the critical role of TrkB and BDNF communication in HD pathogenesis, the vulnerability of cultured HD neurons to BDNF withdrawal has been extensively studied [57, 62–64]. Indeed, in our study, we detected extensive death, by Annexin V staining, of neurons derived from HD-NPCs upon BDNF withdrawal, which was ameliorated by HTT knockdown (Fig. 4M, N). Similarly, MSNs exhibited the maximal sensitivity (Fig. 4M, N), echoing their repressed expression of the TrkB receptor. Overall, we demonstrated that generated MSNs more accurately recapitulate the pathologic features of HD.

### The propensity of polyQ aggregations in MSNs

**Fig. 5:**
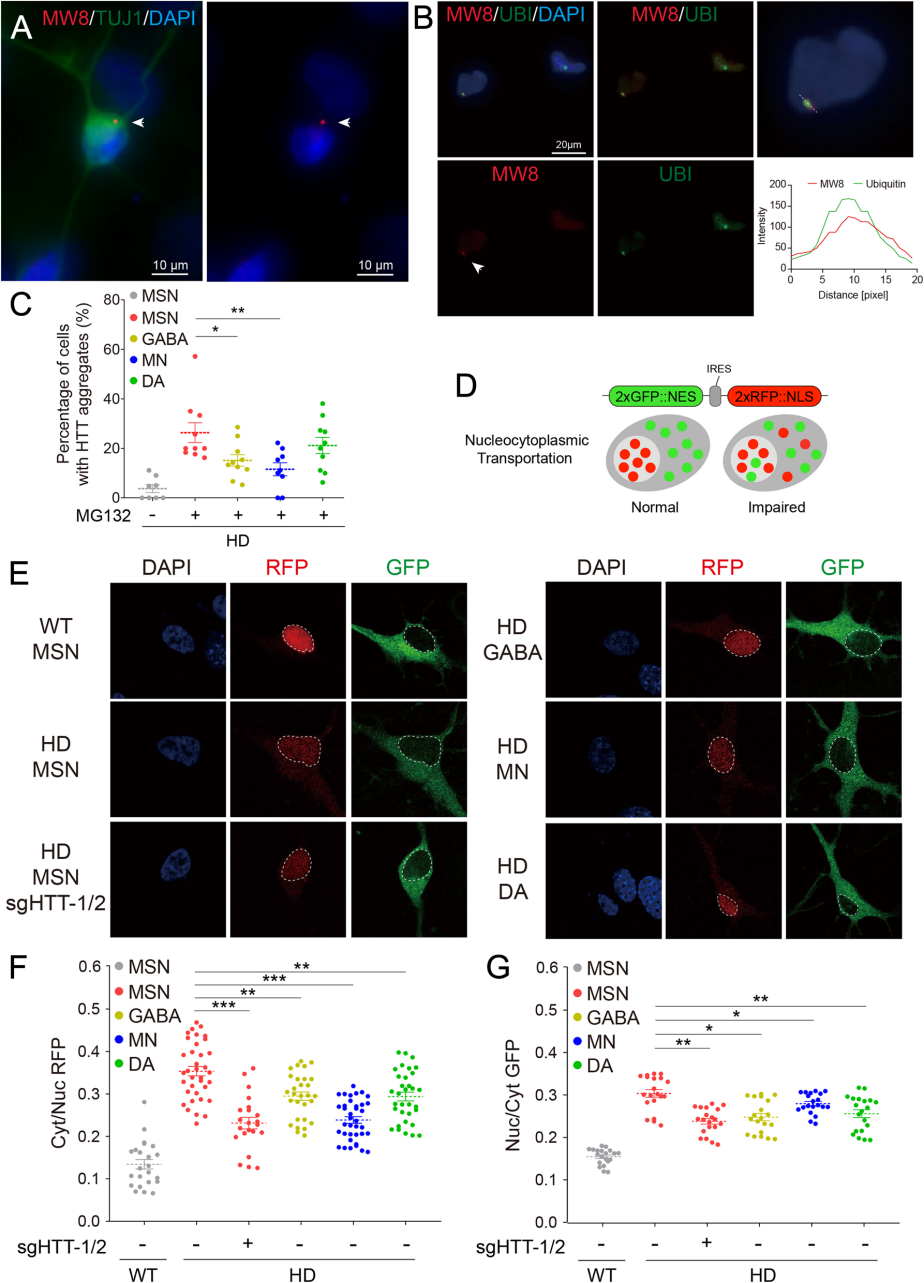
The propensity of polyQ aggregations and NCT disturbance in HD-MSNs. **A**–**C** The polyQ aggregates were detected by immunostaining of HTT (MW8) and ubiquitin in differentiated neurons on the glia layer. The treatment with MG132 was applied to trigger the formation of aggregates (arrowheads). Scale bars, 10 μm and 20 μm. **D** The 2Gi2R reporter system containing 2xGFP::NES-2xRFP::NLS tracks protein translocation inside and outside the nucleus. **E**–**G** The NCT disturbance in differentiated neurons on the glia layer was assessed by the ratios of nucleic GFP (Nuc/Cyt GFP) and cytoplasmic RFP (Cyt/Nuc RFP), increasing of which indicates an impaired NCT. *p < 0.05; **p < 0.01; ***p < 0.001

Due to the rejuvenated process during iPSC generation [39, 65], the number of mHTT aggregates in hPSC-derived cells is much lower than in aged adult HD cells. Given this prerequisite, we tested whether the propensity for polyQ aggregation would vary among different neural subtypes. We indeed observed considerably fewer aggregates in naive MSNs, and treatment with one of the proteasome inhibitors, MG132, further triggered aggregation, resulting in more than 20% of the cells showing aggregation (Fig. 5A–C). However, in other neural subtypes, the same treatment only led to aggregation in up to 20% of cells (Fig. 5C). Moreover, the MW8-stained mHTT aggregates co-localized with ubiquitin (Fig. 5A, B), hinting at possible aggregate clearing by the ubiquitin–proteasome system (UPS).

### Nucleocytoplasmic transport disturbance in HD-MSNs

Nucleocytoplasmic transport (NCT) is a process of bidirectional transport of biological macromolecules between the nucleus and the cytoplasm. It is the basic life activity of eukaryotic cells, which is mainly regulated by the nuclear pore complex. In recent years, mounting evidences have revealed that NCT plays an important role in the physiology of aging and multiple neurodegenerative diseases, including HD, but through distinct mechanisms [66].

We applied the 2Gi2R reporter system containing 2xGFP::NES-2xRFP::NLS to track protein translocation inside and outside the nucleus (Fig. 5D). Due to the fusion of NES (nuclear export signal) and NLS (nuclear localization signal), GFP and RFP are expected to be precisely localized in the cytoplasm and nucleus, respectively. In WT-MSNs, both GFP and RFP signals were concentrated in the correct regions (Fig. 5E). However, in HD neurons, increased ratios of nucleic GFP (Nuc/Cyt GFP) and cytoplasmic RFP (Cyt/Nuc RFP) were observed (Fig. 5E–G), suggesting that the protein NCT is impaired. Similarly, the NCT disturbance was most prominent in HD-MSNs, compared to other HD neural subtypes, which can be reversed by HTT knockdown (Fig. 5E–G).

### LMNB1 mislocalization and hypofunction in HD-MSNs

**Fig. 6:**
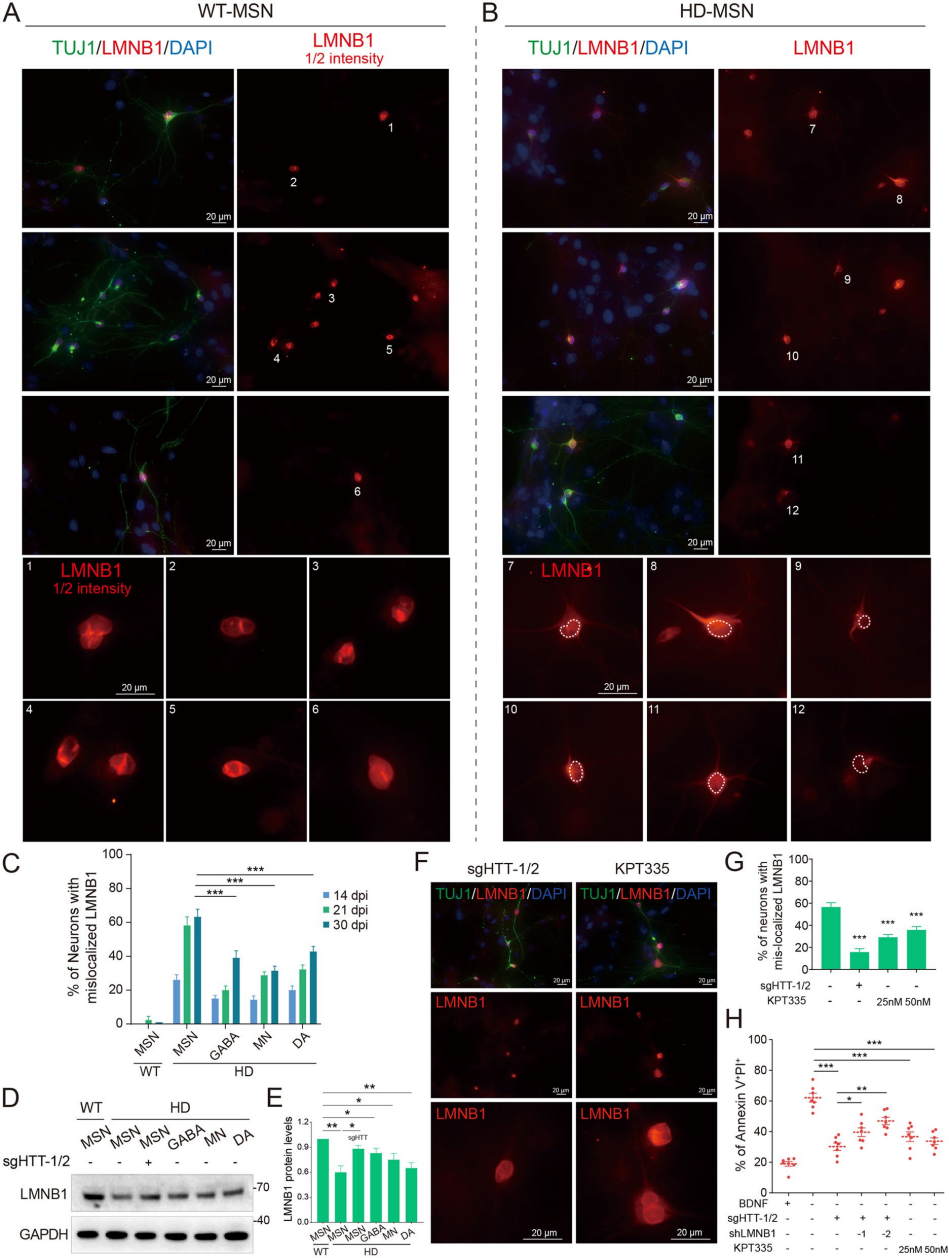
LMNB1 mislocalization and hypofunction in HD-MSNs. **A**–**C** Both WT and HD neurons on the glia layer were immunostained by LMNB1. Prominent LMNB1 mislocalizations into the cytoplasm were observed in HD neurons. Dashed circles denote nucleus. Scale bars, 20 μm. **D**, **E** LMNB1 levels were detected by immunoblot in differentiated neurons. **F**, **G** HTT knockdown or KPT335 treatment effectively ameliorated the LMNB1 mislocalization. **H** HTT knockdown or KPT335 treatment attenuated neuronal death upon BDNF withdrawal. However, the therapeutic effects of HTT knockdown were partially diminished by LMNB1 knockdown. *p < 0.05; **p < 0.01; ***p < 0.001

Given the apparent NCT impairment found in HD neurons, we further asked whether the nuclear biology of HD neurons was changed. To this end, we performed immunostaining on generated neurons using LMNB1 antibody. Unexpectedly, we found that the LMNB1 intensity was much weaker in HD-MSNs than that in WT-MSNs, which was reminiscent of a possible downregulation of LMNB1 protein levels (Fig. 6A, B). Nonetheless, we revealed a striking yet previously unappreciated finding that LMNB1 was mislocalized to the cytoplasm and further extended to the axons of HD neurons, since the early stages of neural differentiation (Fig. 6A, B). The proportion of LMNB1 mislocalization reached more than 60% in MSNs at 21- and 30-days post infection (dpi), compared with around 20 ~ 40% in other neural subtypes (Fig. 6C). This may be partly related to the NCT disturbance, as the transportation of LMNB1 might be impaired.

While LMNB1 is mislocalized to the cytoplasm in HD neurons, the decrease in nuclear lamina intensity could also be the result of protein re-localization without compromising the total amount of LMNB1 protein. To address this, we did immunoblot to assess LMNB1 protein levels. The results, however, showed a most remarkable reduction of LMNB1 levels in HD-MSNs (Fig. 6D, E), thus reinforcing the notion of LMNB1 hypofunction.

### Targeting NCT disturbance mitigated LMNB1 hypofunction and neuronal death

To further investigate the effects of LMNB1 hypofunction, we also employed shRNAs knockdown of LMNB1 (Fig. S9B), as well as sgRNAs knockdown of HTT, while assessing the neuronal death upon BDNF withdrawal. Importantly, we found that HTT knockdown effectively ameliorated the LMNB1 mislocalization and restored the LMNB1 protein level in MSNs (Fig. 6D–G). We previously demonstrated that knockdown of HTT attenuated neuronal death (Fig. 4M, N); however, this process was suppressed by LMNB1 knockdown, suggesting that the therapeutic effects of sgHTT were partially diminished by LMNB1 hypofunction (Fig. 6H).

In addition to genomic targeting of HTT, chemical intervention in NCT disturbance is also a promising therapeutic approach. Considering the requirement of clinical translatability, we chose KPT335, which is an orally selective inhibitor of XPO1/CRM1-dependent nuclear export; XPO1 is one of the most important receptors responsible for the nuclear transport of various proteins and RNAs [67]. Indeed, after treatment with KPT335 at varied concentrations for 24 h, the NCT disturbance was greatly attenuated in MSNs (Fig. 6F, G). However, the LMNB1 protein level was not significantly restored (Fig. S9C, D), probably due to that the KPT335 treatment time was rather short or that XPO1-mediated transportation might be less related with the LMNB1 regulation machinery. Nevertheless, the treatment also attenuated neuronal death caused by BDNF withdrawal (Fig. 6H), and ameliorated the reduced morphological complexity of HD neurons (Fig. S9E). We assume that the adequate LMNB1 level in the nucleus might be the key. LMNB1 knockdown or mislocalization might cause a reduction of the nucleus LMNB1 level, which thus plays a pivotal role in the neural survival. Taken together, we validated that targeting the NCT disturbance, both genetically and pharmacologically, could effectively ameliorate neuronal death, which may provide a promising therapeutic intervention for treating HD.

## Discussion

**Fig. 7:**
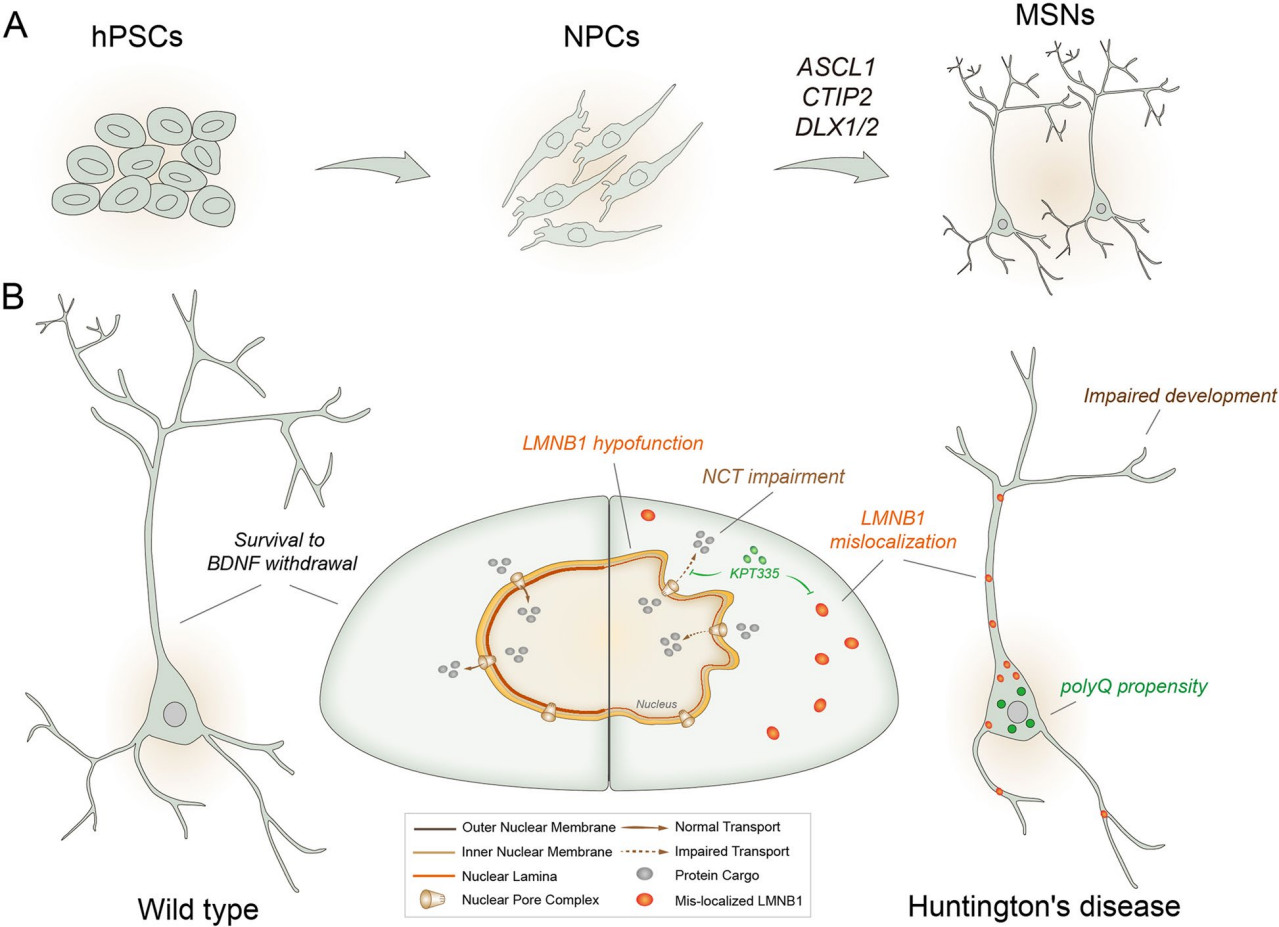
Proposed model. **A** This study established a method that can rapidly and efficiently generate MSNs, by introducing a certain set of transcription factors, ASCL1/CTIP2/DLX1 or DLX2. **B** This study compared the capacity of four major neural subtypes including MSNs, in parallel, in modeling HD. Of note, MSN mimics the pathological features of HD more readily than other neural subtypes in a number of ways. For instance, MSNs exhibited stronger polyQ aggregation propensity and overexpression induced neurotoxicity, more severe BDNF/TrkB signaling dysfunction, greater susceptibility to BDNF withdrawal, and more severe NCT disturbances. This study further revealed that the nuclear lamina protein LMNB1 was greatly reduced in HD neurons and mislocalized to the cytoplasm and further extended to axons. Knockdown of HTT or treatment with KPT335, an orally SINE, effectively attenuated the pathological phenotypes and alleviated neuronal death caused by BDNF withdrawal. This study thus establishes an efficient method for obtaining MSNs and underscores the necessity of using high-purity MSNs to study HD pathogenesis, as MSNs performed best in mimicking multiple pathological features. On this basis, the MSN cellular platform shows increased potential for drug/chemical screening for the treatment of HD as well as for a variety of high-throughput analyses, such as multi-omics, mass spectrometry, next-generation sequencing, and biochemical assays

In this study, we screened a certain set of transcription factors, ASCL1/CTIP2/DLX1, that rapidly and efficiently generate MSNs (over 90%) within 21 days (Fig. 7). We simultaneously compared the ability of four major neural subtypes, including MSNs, in modeling HD. Of note, compared to other neural subtypes, MSNs exhibited higher polyQ aggregation propensity and overexpression induced neurotoxicity, more severe dysfunction in BDNF/TrkB signaling, greater susceptibility to BDNF withdrawal, and more severe NCT disturbances (Fig. 7).

### The necessity and applications of high-purity MSNs

Our results underscore the necessity of using high-purity MSNs to study HD pathogenesis especially the MSN-selective vulnerability, which performs best in mimicking multiple pathological features. On this basis, the MSN cellular platform shows increased potential for drug/chemical screening for treating HD, as well as for various high-throughput analyses, such as multi-omics, mass spectrometry, next-generation sequencing, and biochemical assays. Another application of this research may be to provide an “unlimited” source of cell therapy, aiming to replenish damaged neurons and re-establish neural projections. However, in order to achieve clinical translation, our strategy of MSN differentiation must be further adapted, by replacing gene delivery with low immunogenicity adeno-associated viruses (AAVs) and employing a well-established clinical-grade differentiation system.

Given that different neural subtypes are obtained from the same starting donor, our strategy manifests extra advantages. For instance, it allows the comparison of changes in the expression profiles of different neural subtypes, including MSNs, by high-throughput omics detection. This facilitates us to systematically probe potential new mechanisms of selective death of MSNs during the development of HD. Second, it can be used to construct co-culture systems to mimic defined neural circuits in a dish. As a canonical approach, excitatory glutamatergic neurons were co-cultured with inhibitory GABAergic neurons to test the establishment of functional synaptic connections [68]. The ratio of co-cultured neurons was set at GABA (20%) and GLUT (80%), similar to that in mammalian cortical networks. In deciphering the pathogenesis of neurological diseases, other neural circuits can also be studied. Interestingly, different neural subtypes, induced by transcription factors, can be labeled with diverse tags (Flag/Myc), or colors (GFP/mScarlet), which facilitates the study of neuronal interactions by a variety of assays such as electrophysiology detection, cell sorting, or biochemical dissection, in a subtype-specific manner.

Our study can be further refined, as the current protocol does not differentiate between MSN subtypes. Given that D1 and D2 MSNs have important but distinct roles [69], it is desirable to generate specific MSN subtypes from hPSCs. For this purpose, the introduction of other factors in addition to ASCL1/CTIP2/DLX1 is required. Among the potential candidates, SP9, a zinc finger transcription factor that is necessary for the maintenance and survival of striatopallidal MSNs [70], might be an optimal choice.

### The pathological alterations modeled by MSNs

The NCT disturbance was seen in previous studies on varied HD models [58, 59], which is shown to be most prominent in MSNs, compared to other subtypes. Our study provides another evidence that the NCT disturbance is becoming a shared hallmark in the pathogenesis of multiple neurodegenerative diseases, such as AD, ALS, HD, and NIID, among others [66, 71]. Mechanistically, pathogenic proteins (mutated TDP-43, Tau, FUS, HTT) or RNAs (C9orf72 G4C2 hexanucleotide repeats) form aggregates or deleterious RNA G-quadruplexes, and further bind and sequester nucleoporins and nuclear transporters [58, 59, 71–74]. Notably, this disturbance is aggravated with age [58, 59]. However, the types of nucleoporins and their interactions with pathogenic proteins/RNAs are not the same in different diseases [66], which reveals the diversity of mechanisms involved in the development of NCT disorders.

LMNB1 hypofunction and mislocalization were rarely discovered in HD. Interestingly, they appeared to be neuron-specific and were barely seen in HD-NPCs (Fig. S8) and HD-iPSCs (data not shown). The occurrence of nucleus dysfunctions sounds reasonable, since LMNB1 has been tightly associated with neural development [75–79], and also gene regulation by chromatin-associated mechanisms [80, 81]. The pathology of LMNB1 has also been studied in other neurodegenerative diseases. For example, the nuclear-envelope impairment was found in the brain tissue of patients with clinically diagnosed PD [82]. During differentiation of neural stem cells (NSCs) from PD-iPSCs harboring LRRK2^G2019S^ mutation, nuclear aberrations progressively increased and led to compartmentalized pedal-like nuclei [82]. Local loss of LMNB1 was found in certain nuclear-envelope micro-domains. Thus, our findings partially coincide with the results of that study.

While LMNB1 decrease is an important hallmark of aging [83], it must be cautious to conclude whether the pathology is sorely due to aging or the pathogenic genes, particularly in aging-related neurodegenerative diseases. To address this, endeavors have devoted and revealed that nuclear-architecture defects could be rescued by the inhibition of LRRK2 kinase activity, or the correction of LRRK2^G2019S^ mutation [82]. Similarly, in our study, knockdown of HTT (including mHTT) ameliorated nuclear aberrations and NCT disturbance. These demonstrate that LMNB1 hypofunction plays a pathogenic role in the development of neurodegenerative diseases.

In a recent study from our group, we found similar defects in nuclear architecture, as well as NCT disturbance, in modeling DYT1 dystonia [84]. LMNB1 mislocalization was also observed in both DYT1-iPSC derived MNs and directly reprogrammed MNs from human adult fibroblasts. However, LMNB1 levels were upregulated that led to a thickened, probably more rigid, nuclear lamina. This is contrary to our findings in HD. Thus, the results of our two studies suggest that maintaining LMNB1 homoeostasis is important for normal neuronal physiology.

Given that disturbed NCT is an important pathological manifestation of HD neurons, we may be able to ameliorate their neuropathological changes by targeting NCT. Our previous study found that knockdown of LMNB1 could alleviate, to some extent, the neurodevelopmental impairments in DYT1 due to the NCT disturbance [84]. However, considering that LMNB1 plays an essential function in maintaining the nucleus architecture, and also is required to be at a homeostatic level, targeting LMNB1 alone may not be clinically significant. Alternatively, targeting NCT by small molecule compounds holds promise for modulating the pathology of these diseases. In our study, we tested the potential function of KPT335, an orally selective inhibitor of XPO1/CRM1-dependent nuclear export signaling that has been applied in treating many other diseases. Excitingly, KPT335 effectively suppressed the NCT disturbance, including LMNB1 mislocalization, and attenuated neuronal death caused by BDNF withdrawal. Thus, this may provide an alternative therapeutic avenue for the treatment of HD.

## Limitations of the study

This study has several limitations. First, we recognized striatal MSNs as the primary damaged neurons for study; however, neurons in the cortex are also affected in HD [61]. In particular, BDNF/TrkB signaling was studied in both cortical and striatal neurons. Therefore, further studies will also need to assess if similar pathological changes of MSNs would be observed in cortical neurons. Second, hPSCs undergo a rejuvenation process during reprogramming, which renders hPSCs and derived neurons in a virtually youthful state. It is an extra yet common challenge for hPSCs in modeling aging-related neurodegenerative diseases. It might be somewhat addressed by serial passaging, or by introducing senescence factors, such as overexpression of progerin [65] and telomerase manipulation [85]. Third, although we observed a deleterious effect of LMNB1 hypofunction and mislocalization, the line-to-line variability of different HD-hPSC lines as well as the underlying mechanisms of the NCT disturbance require further investigations.

### Supplementary Information

**Additional file 1: Fig. S1** The overall schedule of neural differentiation from hNPCs. **Fig. S2**. Tests of neural induction capacities. **Fig. S3**. Induction of proneural factors in non-neural cells. **Fig. S4.** Screening for MSN lineage factors working with ASCL1 in synergy. **Fig. S5.** Characterization of induced neurons. **Fig. S6.** High MOI of viruses led to dramatic neuron death. **Fig. S7.** Knockdown of HTT by sgRNAs. **Fig. S8.** Neural differentiation from hPSCs. **Fig. S9.** Neural survival and LMNB1 levels. **Table S1.** Primer sequences for quantitative PCR. **Table S2.** Antibody list.

## Data Availability

Data were available upon request from the corresponding authors.
