# Supplementary material for: Rapid and high-purity differentiation of human medium spiny neurons reveals LMNB1 hypofunction and subtype necessity in modeling Huntington’s disease
Source: Inflamm Regen. 2024 Feb 15;44:7. doi: 10.1186/s41232-024-00320-x (PMC10870681; doi:10.1186/s41232-024-00320-x)
Supplement: Supplementary file 1 — Additional file 1: Fig. S1 The overall schedule of neural differentiation from hNPCs. Fig. S2. Tests of neural induction capacities. Fig. S3. Induction of proneural factors in non-neural cells. Fig. S4. Screening for MSN lineage factors working with ASCL1 in synergy. Fig. S5. Characterization of induced neurons. Fig. S6. High MOI of viruses led to dramatic neuron death. Fig. S7. Knockdown of HTT by sgRNAs. Fig. S8. Neural differentiation from hPSCs. Fig. S9. Neural survival and LMNB1 levels. Table S1. Primer sequences for quantitative PCR. Table S2. Antibody list. [file 41232_2024_320_MOESM1_ESM.docx]

**Supplementary Materials**

**Supplementary Figures**


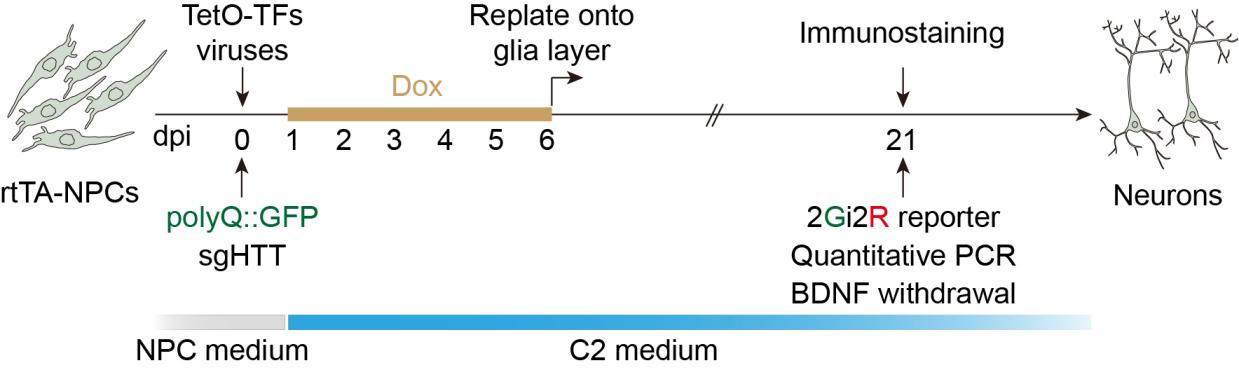


**Fig. S1 The overall schedule of neural differentiation from hNPCs.**

**
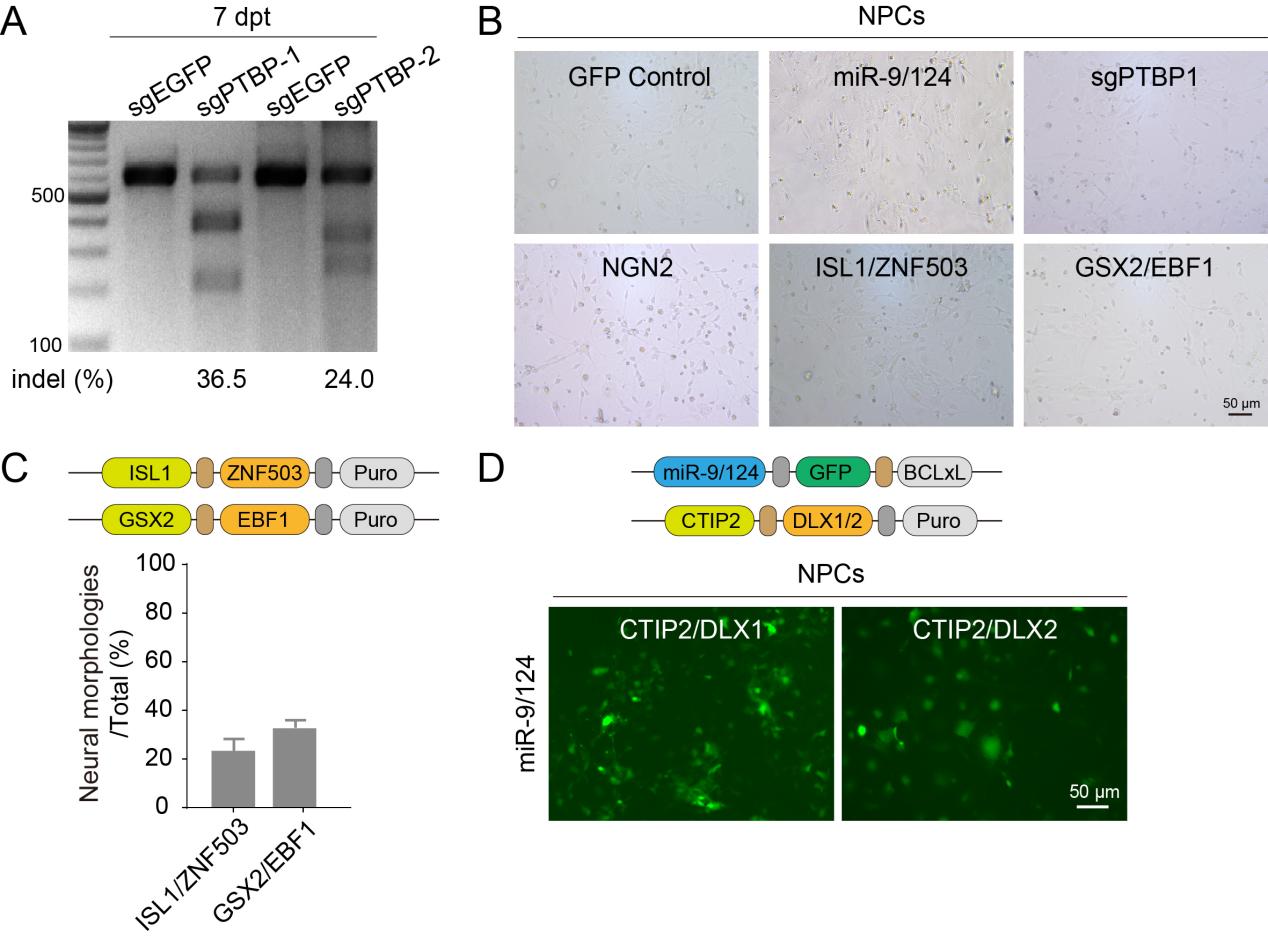
**

**Fig. S2 Tests of neural induction capacities.** (A) The T7E1 assay was performed to examine the genomic cleavage efficacy of sgPTBP1s in 293T cells. (B, C) The combination of MSN lineage factors, including ISL1/ZNF503 and GSX2/EBF1, without adding proneural factors, was tested to induce MSNs. Neither group was effective in inducing neural morphologies. Scale bar, 50 μm. (D) miR-9/124, together with CTIP2 and DLX1/2, were applied to induce cells. Scale bar, 50 μm.

**
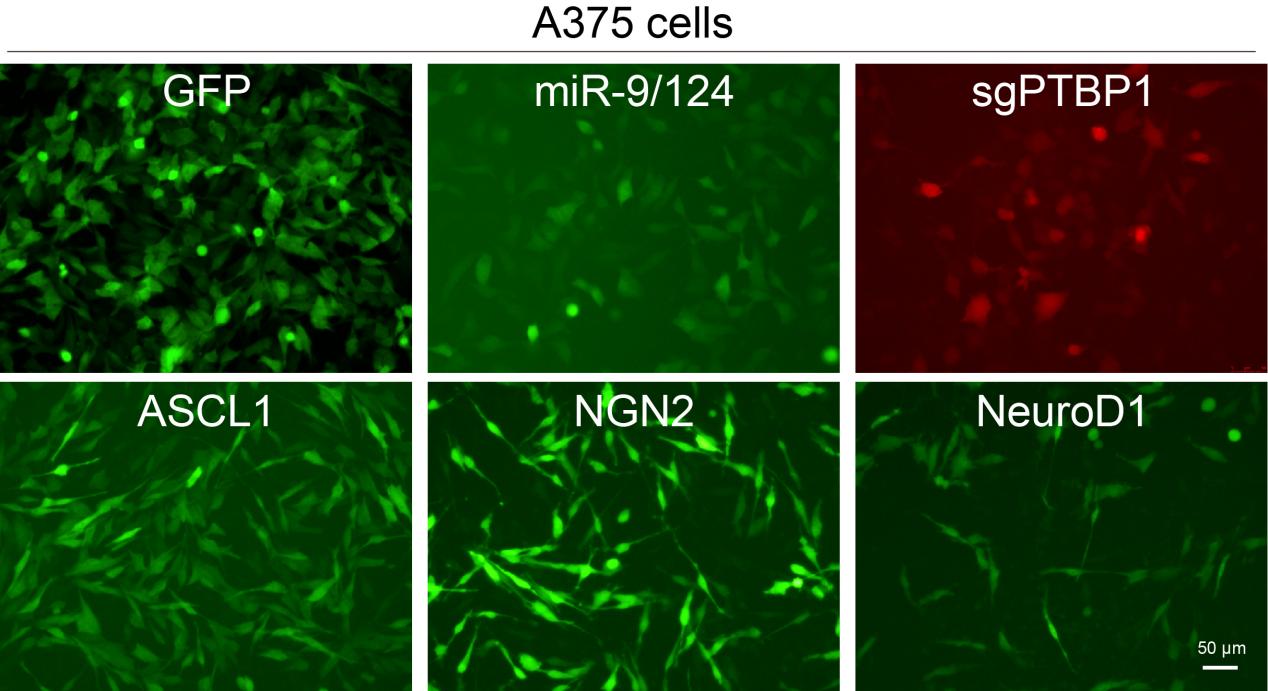
**

**Fig. S3 Induction of proneural factors in non-neural cells.** NGN2 is better at initiating cellular polarity in non-neural A375 cells, and triggers morphology similar to neural processes. Scale bar, 50 μm.

**
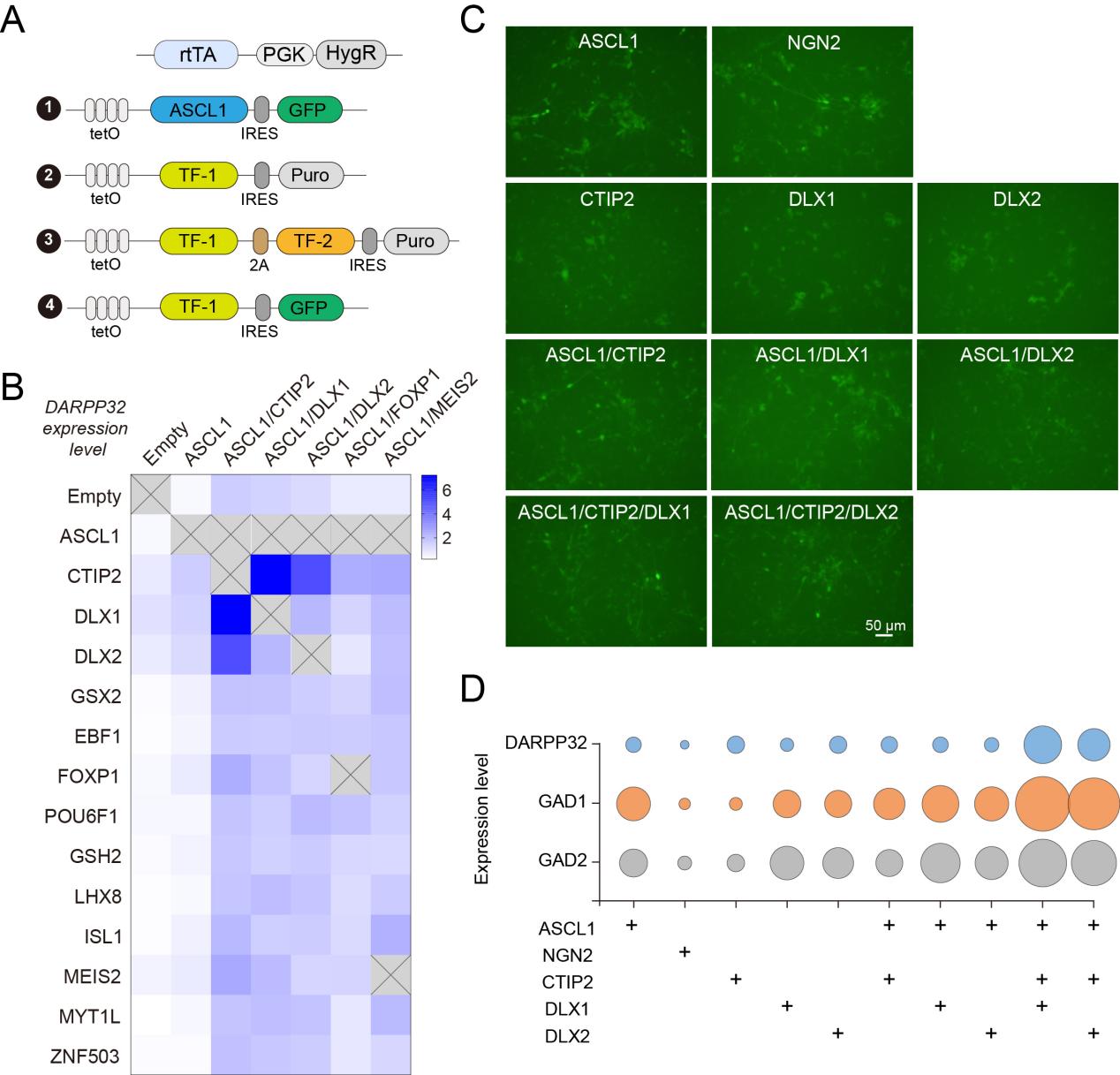
**

**Fig. S4 Screening for MSN lineage factors working with ASCL1 in synergy.** (A) The construction strategy of using MSN lineage factors. Different combinations of transcription factors were induced expressed in rtTA-expressing stable cells. (B, C) Expression of DARPP32, a major marker of MSN, was detected by quantitative PCR. In particular, ASCL1 worked well with CTIP2 and DLX1/2 to efficiently induce DARPP32 expression, whereas neither ASCL1 nor any two of these transcription factors alone induced DARPP32 expression, albeit causing neural morphology changes. Scale bar, 50 μm. (D) Quantitative expression analysis of ASCL1/CTIP2/DLX/1/2 induced neurons. The ASCL1/CTIP2/DLX/1/2 groups induced the expression of GABAergic markers, such as GAD1 and GAD2, which was also seen in groups containing ASCL1 or DLX1/2. The NGN2 control group did not induce the expression of GABAergic markers.

**
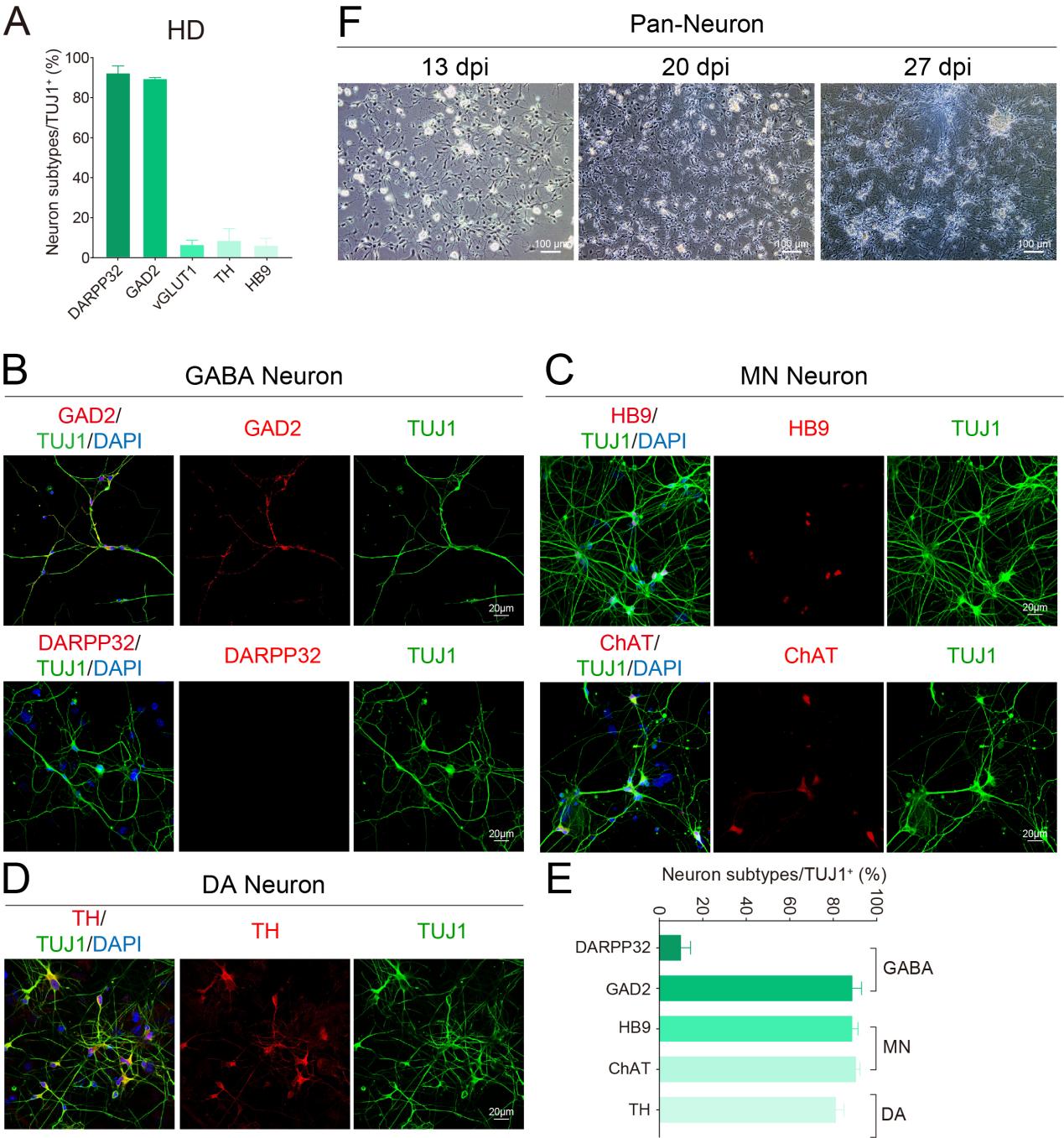
**

**Fig. S5 Characterization of induced neurons.** (A) MSNs were differentiated from HD diseased cells. At 21 dpi, neuron identities were examined by immunostaining of typical markers. Compared to that in WT cells, the efficiency of MSN differentiation in HD cells was not compromised. (B-E) Neuron identities of other neural subtypes, including GABA, MN and DA, were examined by immunostaining of typical markers at 21 dpi. Scale bars, 20 μm. Similarly, over 80% of induced neurons were positive for GAD2, HB9/ChAT and TH, respectively. (F) NPCs were also differentiated towards pan-neurons, as a mixed neuron control, without adding transcription factors. Bright-field pictures were taken at 13, 20, 27 dpi respectively. Based on the morphologies, pan-neurons were efficiently generated. Scale bars, 100 μm.

**
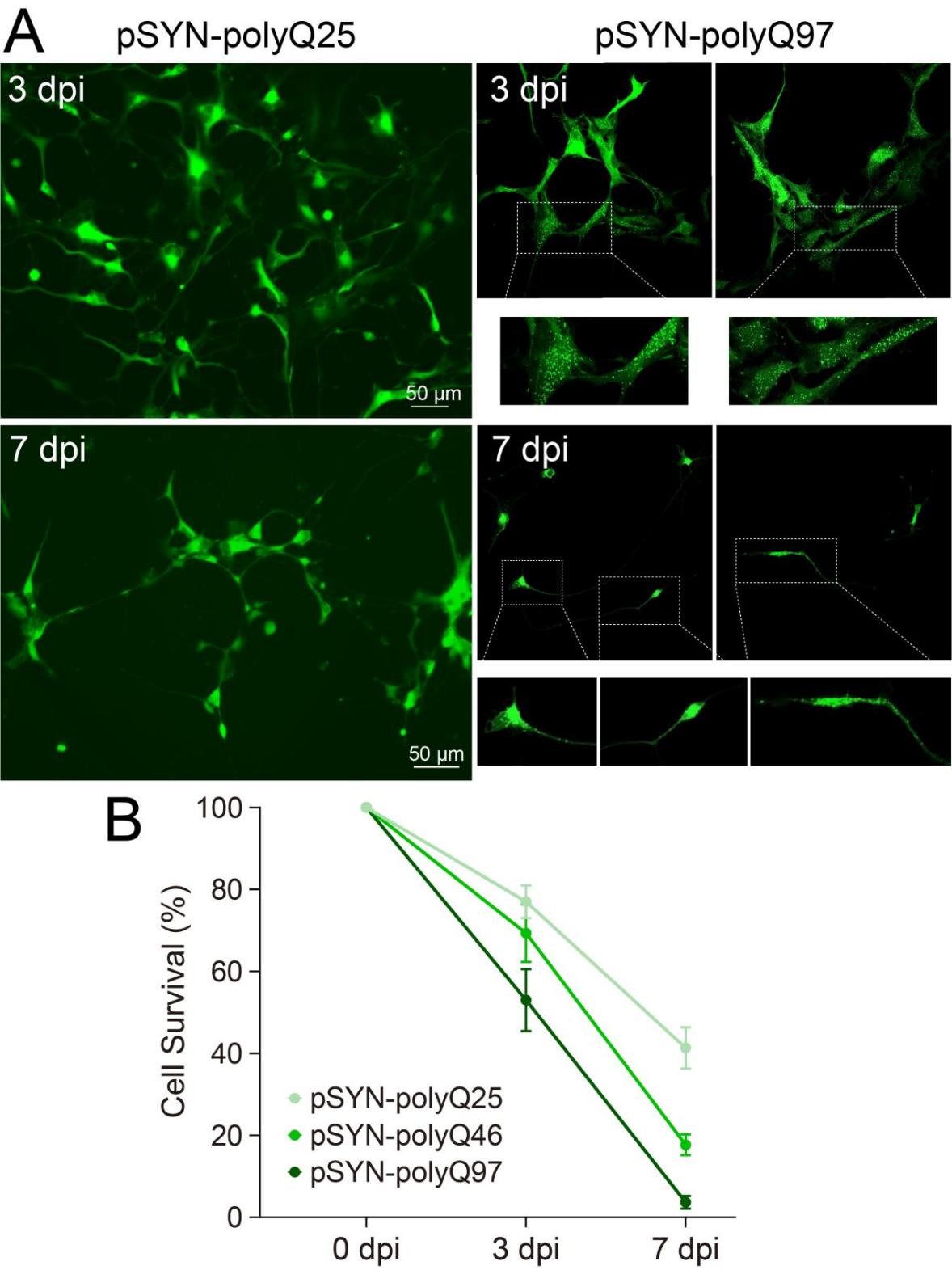
**

**Fig. S6 High MOI of viruses led to dramatic neuron death.** High MOI of polyQ::GFP overexpression caused extensive aggregates, particularly in the polyQ97::GFP group, and led to dramatic neuronal death within 7 days. Scale bars, 50 μm.

**
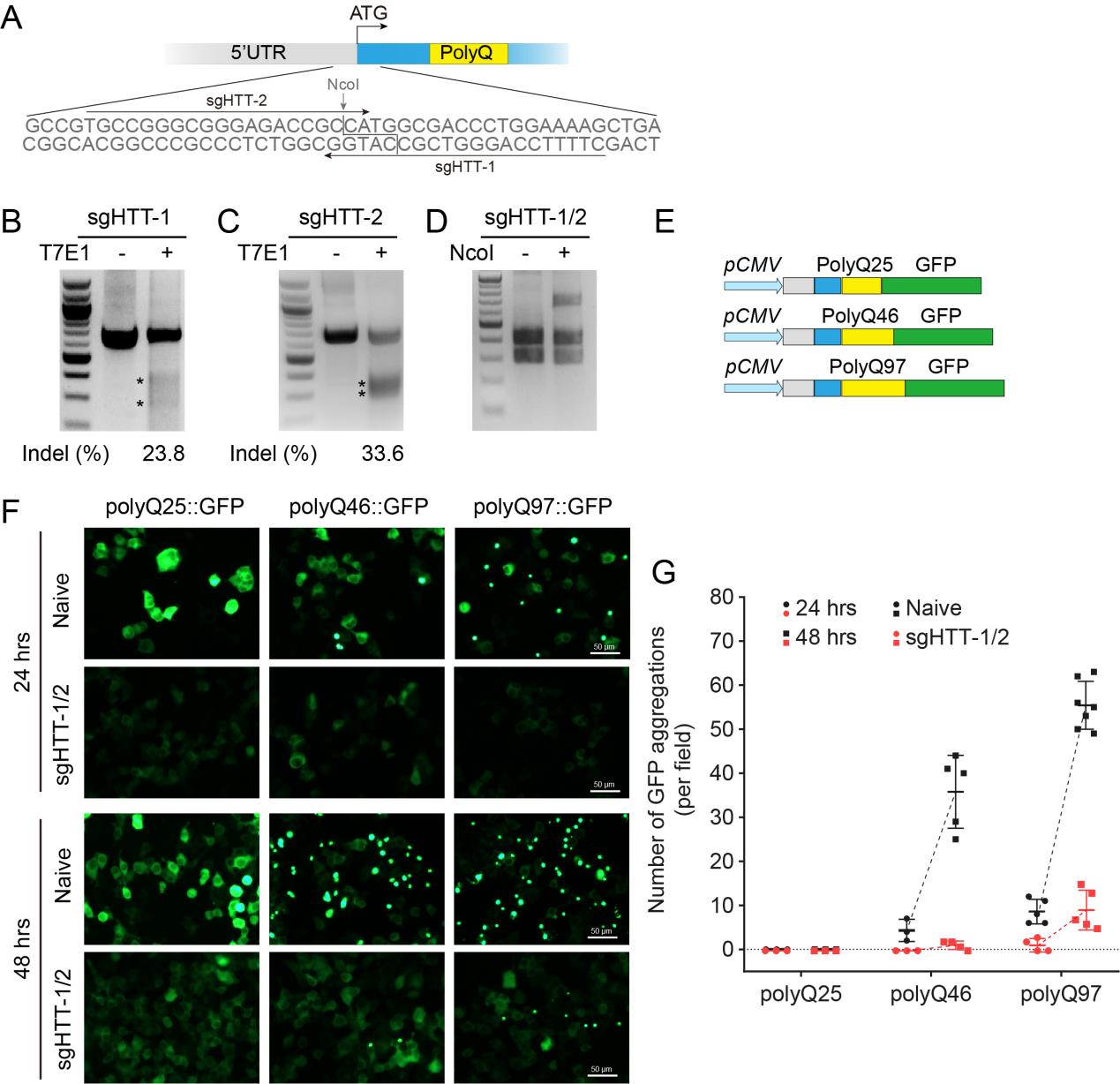
**

**Fig. S7 Knockdown of HTT by sgRNAs.** (A) Two sgHTTs (sgHTT-1 and sgHTT-2) were designed to target genomic DNAs flanking the start codon of HTT CDS. (B-D) The cleavage efficiencies of the sgHTTs on genomic DNA were assessed by T7E1 assay and NcoI digestion. (E) Different lengths of polyQs::GFP (Q25, Q46, Q97) driven by the CMV promoter were respectively constructed into vectors. (F, G) The targeting effect of sgHTTs was further examined *in vivo*. Vectors of polyQs::GFP and sgHTTs were simultaneously transfected into 293T cells. After 24 and 48 hrs, the number of polyQ::GFP aggregates was recorded and analyzed, respectively. Scale bars, 50 μm.

**
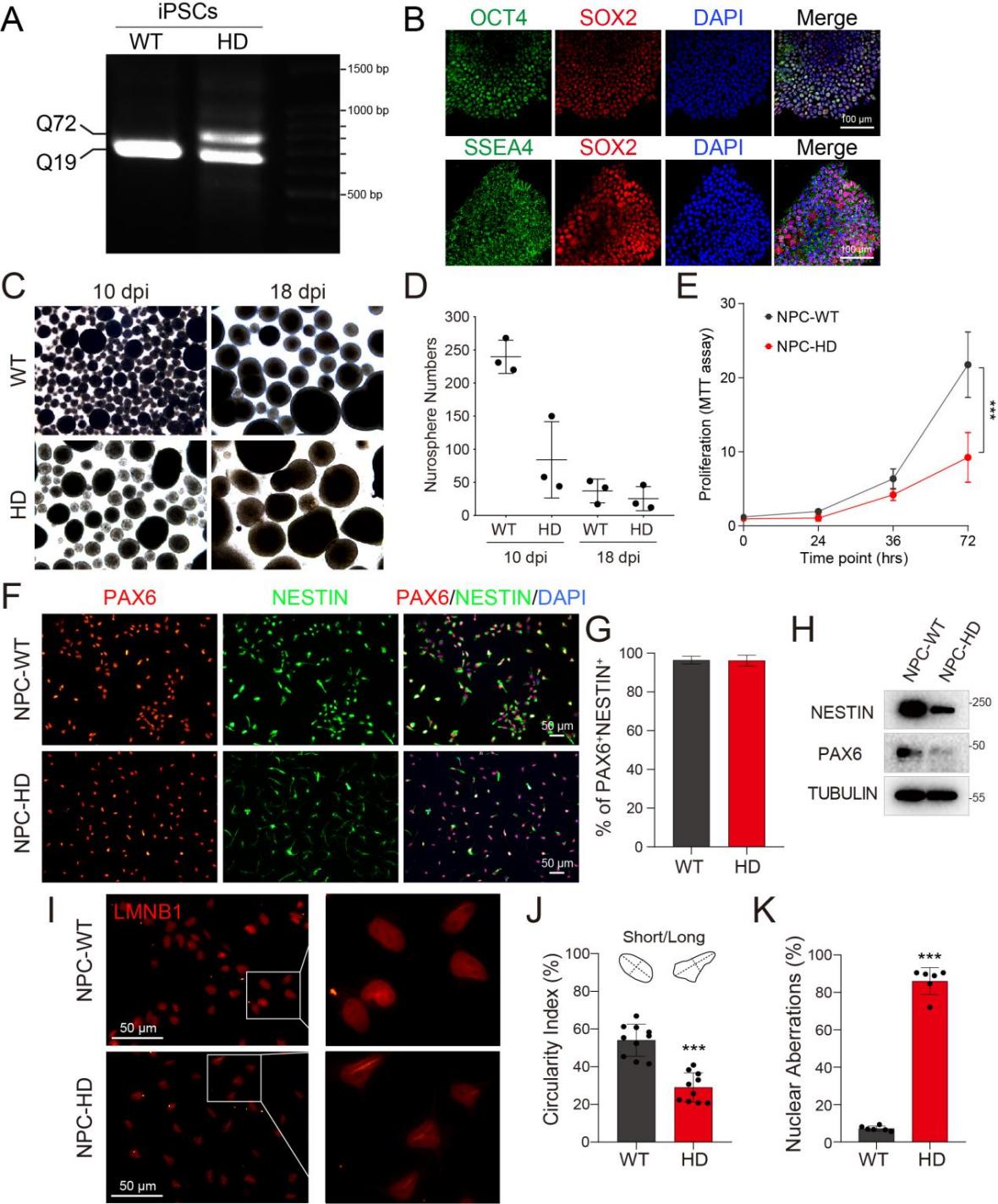
**

**Fig. S8 Neural differentiation from hPSCs.** (A, B) One line of HD-iPSC containing heterogeneous polyQ^19/72^, which are positive for hPSC markers including OCT4, SOX2 and SSEA4, was used. Scale bars, 100 μm. (C, D) Both WT- and HD-iPSCs were differentiated by dual-SMAD inhibition, followed by suspension culture as neurospheres and eventually monolayer culture as NPCs. (E) The proliferation rates of NPCs were assessed by MTT assay. (F, G) Immunostaining was performed to detect NPC markers, such as PAX6 and NESTIN. The yield of NPCs (more than 96% of PAX6^+^ NESTIN^+^) from the HD-iPSC is comparable to that from the WT-iPSC. Scale bars, 50 μm. (H) Immunoblotting was performed to detect NPC markers. The levels of PAX6 and NESTIN were lower in HD-NPCs. (I-K) The nuclear morphology of NPCs was detected by immunostaining. HD-NPCs exhibited deformed/aberrant nuclei with a decrease in nuclear circularity. Scale bars, 50 μm. ***, p < 0.001.

**
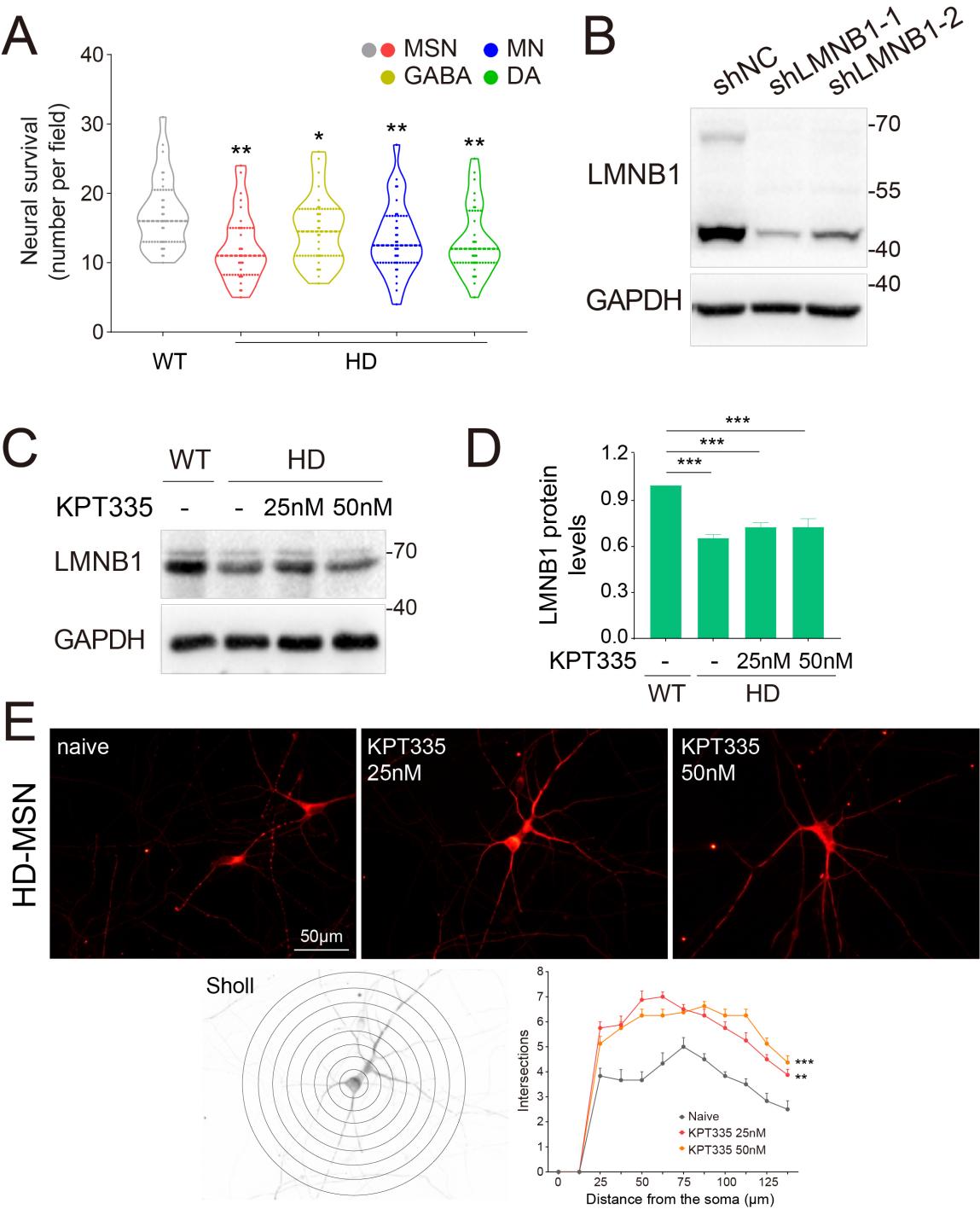
**

**Fig. S9 Neural survival and LMNB1 levels.** (A) Neural survival in WT and HD neurons. (B) Confirmation of LMNB1 knockdown by shRNAs. (C, D) LMNB1 protein levels in MSNs after KPT335 treatment. (E) The morphological complexity of HD-MSNs after KPT335 treatment was assessed by Sholl analysis. Scale bar, 50 μm. *, p < 0.05; **, p < 0.01; ***, p < 0.001.

**Supplementary Tables**

**Table S1 Primer sequences for quantitative PCR**

| Gene Name | Sequences |
| --- | --- |
| AADC-F | ACGCAAGTGAATTCCGAAGGAGAG |
| AADC-R | CAGCCGATGGATCACTTTGGT |
| ALDH1A1-F | TTTGGAAGATAGGGCCTGCACTG |
| ALDH1A1-R | CCTGGATGCGGCTATACAACACTG |
| BDNF-F | TGGCTGACACTTTCGAACAC |
| BDNF-R | ATCACCCTGGACGTGTACAA |
| CalR-F | GATCCTGCCAACCGAAGAGAAC |
| CalR-R | CGATGTAGCCACTCCTGTCTGT |
| ChAT-F | GCACTCCAGCTCCTTCAC |
| ChAT-R | CACTGCACCAGGACGATG |
| CHRM4-F | CTCCATGAACCTCTACACCGTG |
| CHRM4-R | GACGCAGAAGTAGCGGTCAAAG |
| DARPP32-F | CCTGAAGGTCATCAGGCAGT |
| DARPP32-R | GGTCTTCCACTTGGTCCTCA |
| DRD1-F | TGGTCTGTGCTGCCGTTATCAG |
| DRD1-R | CAATCTCAGCCACTGCCTTCCA |
| DRD2-F | CAATACGCGCTACAGCTCCAAG |
| DRD2-R | GGCAATGATGCACTCGTTCTGG |
| EN1-F | CGTGGCTTACTCCCCATTTA |
| EN1-R | TCTCGCTGTCTCTCCCTCTC |
| FOXP1-F | CTACCGCTTCCATGGGAAATC |
| FOXP1-R | CTGTTGTCACTAAGGACAGGG |
| GABRA1-F | CACAAGTCTCCTTCTGGCTCAAC |
| GABRA1-R | GGAGTTTCTGGCACTGATGCTC |
| GABRA4-F | TCCTGGACAGTTTGCTCGATGG |
| GABRA4-R | CAGAAACAGGTCCAAAGCTGGTG |
| GABRB3-F | CAGCCAAGGCAAAGAATGACCG |
| GABRB3-R | ATGCCGCCTGAGACCTCATTCA |
| GABRG2-F | GCACACTCATTGTCGTCCTATCC |
| GABRG2-R | CAATGGTGCTGAGGGTGGTCAT |
| GAD1-F | GCGGACCCCAATACCACTAAC |
| GAD1-R | CACAAGGCGACTCTTCTCTTC |
| GAD2-F | TTTTGGTCTTTCGGGTCGGAA |
| GAD2-R | TTCTCGGCGTCTCCGTAGAG |
| GAPDH-F | GGTCTCCTCTGACTTCAACA |
| GAPDH-R | GTGAGGGTCTCTCTCTTCCT |
| GRIA4-F | GCCTATGGAACACTGGATTCAGG |
| GRIA4-R | GCTCTGCTGATCGCATGTAGGT |
| GRIK1-F | GGATGTATGTGCTCTTAGCCTGC |
| GRIK1-R | GTTTTCCACCACGTCTGAGTCAG |
| GRIN1-F | CCAGTCAAGAAGGTGATCTGCAC |
| GRIN1-R | TTCATGGTCCGTGCCAGCTTGA |
| HB9-F | GCACCAGTTCAAGCTCAAC |
| HB9-R | GCTGCGTTTCCATTTCATCC |
| MEIS2-F | GATGAAAGAGACGGCAGCTCC |
| MEIS2-R | GGGTTGAGGTTGCATCATCG |
| MMP9-F | GCCACTACTGTGCCTTTGAGTC |
| MMP9-R | CCCTCAGAGAATCGCCAGTACT |
| NKX2.1-F | AGCACACGACTCCGTTCTC |
| NKX2.1-R | GCCCACTTTCTTGTAGCTTTCC |
| PENK-F | AATGCAGCCAGGATTGCGCGAC |
| PENK-R | TCTGGTTTGGACAGCTGCAGGA |
| PV-F | TGCAGGATGTCGATGACAGA |
| PV-R | TTTCTTCAGGCCGACCATTT |
| SP9-F | TCGTGTGCAACTGGCTCTTCTG |
| SP9-R | TGTGTTTGCTCAGGTGGTCGCT |
| SST-F | CAAGCCGCTTTAGGAGCGAG |
| SST-R | AGGCGGCAGGACAGCATCT |
| TAC1-F | TTACTGGTCCGACTGGTACGAC |
| TAC1-R | CAAAGAACTGCTGAGGCTTGGG |
| TH-F | GAGTACACCGCCGAGGAGATTG |
| TH-R | GCGGATATACTGGGTGCACTGG |
| TrkB-F | TTTGTACTGCCTGAATGAAAGC |
| TrkB-R | TTTGCAGATGGCAAACCAC |
| VAChT-F | TTCGCCTCTACAGTCCTGTTC |
| VAChT-R | GCTCCTCCGGGTACTTATCG |
| vGLUT1-F | CAGAGTTTTCGGCTTTGCTATTG |
| vGLUT1-R | GCGACTCCGTTCTAAGGGTG |
| vGLUT2-F | GGGAGACAATCGAGCTGACG |
| vGLUT2-R | TGCAGCGGATACCGAAGGA |
| VMAT2-F | TTGGTCTGTTGTTTGCCTCGAAAG |
| VMAT2-R | GGGTCCTTCAGCAGCGTGGTTAG |

**Table S2 Antibody list**

| **Name** | **Supplier** | **Cat #** | **IF Dilution** | **WB Dilution** |
| --- | --- | --- | --- | --- |
| ChAT | Millipore | AB144P | 1:200 |  |
| DARPP32 | Cell Signaling Technology | 2302 | 1:50 |  |
| GAD2 | Cell Signaling Technology | 5843 | 1:100 |  |
| GAPDH | ABclonal | AC033 |  | 1:20000 |
| HB9 | DSHB | 81.5C10 | 1:100 |  |
| LMNB1 | Proteintech | 12987-1-AP | 1:1000 | 1:2000 |
| MW8 | DSHB | MW8 | 1:100 |  |
| NESTIN | Millipore | MAB5326 | 1:400 | 1:1000 |
| OCT4 | Santa Cruz | sc-5279 | 1:200 |  |
| PAX6 | Sigma | HPA030775 | 1:500 | 1:1000 |
| SOX2 | Millipore | AB5603 | 1:200 |  |
| SSEA4 | Millipore | MAB4304 | 1:200 |  |
| SYN1 | Cell Signaling Technology | 5297 | 1:500 |  |
| TH | Millipore | AB152 | 1:800 |  |
| TUBULIN | Sigma | T5168 |  | 1:10000 |
| TUJ1 | Biolegend | 802001 | 1:2000 |  |
| TUJ1 | Biolegend | 801201 | 1:1500 |  |
| UBIQUITIN | ABclonal | A19686 | 1:100 |  |
| vGLUT1 | Synaptic System | 135303 | 1:500 |  |
